# Role of SPAK–NKCC1 signaling cascade in the choroid plexus blood–CSF barrier damage after stroke

**DOI:** 10.1186/s12974-022-02456-4

**Published:** 2022-04-12

**Authors:** Jun Wang, Ruijia Liu, Md Nabiul Hasan, Sydney Fischer, Yang Chen, Matt Como, Victoria M. Fiesler, Mohammad Iqbal H. Bhuiyan, Shuying Dong, Eric Li, Kristopher T. Kahle, Jinwei Zhang, Xianming Deng, Arohan R. Subramanya, Gulnaz Begum, Yan Yin, Dandan Sun

**Affiliations:** 1 Department of Neurology, The Second Hospital of Dalian Medical University, Dalian, 116027 Liaoning China; 2 Department of Neurology, University of Pittsburgh, 7016 Biomedical Science Tower 3, 3501 Fifth Ave., Pittsburgh, PA 15260 USA; 3 Pennsylvania State University, State College, PA USA; 4 Department of Neurosurgery, The Massachusetts General Hospital and Harvard Medical School, Boston, MA USA; 5 Institute of Biomedical and Clinical Sciences, Medical School, College of Medicine and Health, University of Exeter, Hatherly Laboratory, Exeter, EX4 4PS UK; 6 State Key Laboratory of Cellular Stress Biology, Innovation Center for Cell Signaling Network, School of Life Sciences, Xiamen University, Xiamen, Fujian China; 7 Renal-Electrolyte Division, Department of Medicine, University of Pittsburgh School of Medicine, Pittsburgh, PA USA; 8 Research Service, Veterans Affairs Pittsburgh Health Care System, Pittsburgh, PA USA

**Keywords:** Bumetanide, Choroid plexus, H_2_O_2_, Na^+^–K^+^–Cl^−^ cotransporter, SPAK, ZT-1a

## Abstract

**Background:**

The mechanisms underlying dysfunction of choroid plexus (ChP) blood–cerebrospinal fluid (CSF) barrier and lymphocyte invasion in neuroinflammatory responses to stroke are not well understood. In this study, we investigated whether stroke damaged the blood–CSF barrier integrity due to dysregulation of major ChP ion transport system, Na^+^–K^+^–Cl^−^ cotransporter 1 (NKCC1), and regulatory Ste20-related proline-alanine-rich kinase (SPAK).

**Methods:**

Sham or ischemic stroke was induced in C57Bl/6J mice. Changes on the SPAK–NKCC1 complex and tight junction proteins (TJs) in the ChP were quantified by immunofluorescence staining and immunoblotting. Immune cell infiltration in the ChP was assessed by flow cytometry and immunostaining. Cultured ChP epithelium cells (CPECs) and cortical neurons were used to evaluate H_2_O_2_-mediated oxidative stress in stimulating the SPAK–NKCC1 complex and cellular damage. In vivo or in vitro pharmacological blockade of the ChP SPAK–NKCC1 cascade with SPAK inhibitor ZT-1a or NKCC1 inhibitor bumetanide were examined.

**Results:**

Ischemic stroke stimulated activation of the CPECs apical membrane SPAK–NKCC1 complex, NF-κB, and MMP9, which was associated with loss of the blood–CSF barrier integrity and increased immune cell infiltration into the ChP. Oxidative stress directly activated the SPAK–NKCC1 pathway and resulted in apoptosis, neurodegeneration, and NKCC1-mediated ion influx. Pharmacological blockade of the SPAK–NKCC1 pathway protected the ChP barrier integrity, attenuated ChP immune cell infiltration or neuronal death.

**Conclusion:**

Stroke-induced pathological stimulation of the SPAK–NKCC1 cascade caused CPECs damage and disruption of TJs at the blood–CSF barrier. The ChP SPAK–NKCC1 complex emerged as a therapeutic target for attenuating ChP dysfunction and lymphocyte invasion after stroke.

**Supplementary Information:**

The online version contains supplementary material available at 10.1186/s12974-022-02456-4.

## Background

The choroid plexus (ChP), a highly vascularized tissue in each ventricle of the brain, is a main secretory source for producing cerebrospinal fluid (CSF), and simultaneously functions as the blood–CSF barrier to prevent toxic metabolites and immune cells from entering into the central nervous system (CNS) [1, 2]. Like other brain barriers, tight junction proteins (TJs) are present between the adjacent choroid plexus epithelium cells (CPECs) in the ChP blood–CSF barrier and are an essential prerequisite for maintaining barrier integrity [3, 4]. Although this barrier plays an important role in the development, homeostasis, and maintenance of the CNS [5, 6], it remains a relatively understudied brain structure. Impaired ChP–CSF structure and function are implicated in the development of several neurological diseases, including excessive CSF accumulation in posthemorrhagic hydrocephalus [7], infiltration of leucocytes in autoimmune diseases (e.g., multiple sclerosis), neuroinflammation in stroke, morphological changes of CPECs with altered CSF production in Alzheimer’s disease (AD) and other neurodegenerative diseases [8–10]. Moreover, new studies show that the ChP is one of the major infiltration routes for lymphocyte invasion into the post-stroke brains and plays a role in regulating neuroinflammatory responses [11]. However, the cellular and molecular mechanisms underlying ChP structural and functional changes in neurodegenerative diseases are not well understood.

Ion transporters that mediate vectorial ion transport across basolateral and apical membranes of CPECs, accompanied by transport of water through aquaporins, produce approximately 500 ml of CSF per day in adult human brains [1, 12, 13]. The cation-chloride cotransporter family protein Na^+^–K^+^–Cl^−^ cotransporter isoform 1 (NKCC1) is located at the apical membrane of the CPECs [14] and contributes approximately half of the CSF secretion in the adult brains [12]. Activation of NKCC1 via the upstream regulatory serine-threonine kinases, the WNK (Kinase with no lysine (K))- SPAK (Ste20-related proline-alanine-rich kinase)/OSR1(oxidative stress-responsive kinase-1), play crucial roles in regulating cell volume and maintaining ionic homeostasis in the CNS [15]. However, during early postnatal brain development, it has been shown that elevated expression of NKCC1 protein in the CPECs increased CSF K^+^ clearance, cerebral compliance, and reduced circulating CSF in mice [16]. In experimental posthemorrhagic hydrocephalus, toll-like receptor 4 (TLR4)- and nuclear factor kappa B (NF-κB)-dependent inflammatory responses stimulate ChP NKCC1-mediated CSF hypersecretion [7]. Moreover, overstimulation of SPAK–NKCC1 complex activity is well documented to be involved in ischemic brain damage through NKCC1-mediated influx of Na^+^ and Cl^−^, leading to the blood–brain barrier (BBB) damage, cytotoxic edema, and neural excitotoxicity [17, 18]. These findings lead us to investigate whether the SPAK–NKCC1 cascade in the CPECs is altered in stroke brains and has impact on the blood–CSF barrier function and brain inflammation.

We reported here that ischemic stroke stimulated phosphorylation activation of SPAK–NKCC1 cascade at the apical membrane of CPECs, which was associated with upregulation of NF-κB, MMP9 and loss of TJs integrity as well as immune cell infiltration in the ChP. H_2_O_2_-mediated oxidative stress directly activated the SPAK–NKCC1 pathway in either cultured CPECs or cortical neurons. Blocking of the SPAK–NKCC1 pathway with the novel SPAK inhibitor ZT-1a or NKCC1 inhibitor bumetanide protected ChP against the stroke-induced damage and oxidative stress-induced apoptosis. We concluded that pathological stimulation of the SPAK–NKCC1 cascade cause damages of CPECs, disruption of TJs, and immune cell infiltration of the ChP. Thus, the SPAK–NKCC1 cascade emerges as a therapeutic target for ChP dysfunction and brain inflammation after stroke.

## Methods

### Chemicals

Hydrogen peroxide (H_2_O_2_, Cat# H325) and Calcein AM (Cat# C3100MP) were purchased from Thermo Fisher Scientific (Waltham, MA). Bumetanide (BMT, Cat# B3023) and propidium iodide (PI, Cat# P4864) were purchased from Sigma-Aldrich (St. Louis, MO). Free radical scavenger Ebselen (Cat# 70530) was from Cayman Chemical Company (Ann Arbor, MI). Novel SPAK inhibitor ZT-1a was developed as described before [19]. Rabbit anti-pSPAK/pOSR1 (pSer383 SPAK/pSer325 OSR1), rabbit anti-SPAK/OSR1, rabbit anti-pNKCC1 (pThr206) were developed by Dr. Yang at Taiwan National University [20, 21], mouse monoclonal anti-NKCC (T4) was from the Developmental Studies Hybridoma Bank (Iowa City, IA). Novel rabbit anti-pT58 antibody was developed as described before [22], which recognizes residue pT58 in Na^+^–Cl^−^ cotransporter (NCC) and cross-reacts with a nearly identical phospho-epitope in NKCC; in mouse NKCC1 this site includes pThr211 NKCC1 [23]. Collagenase type IV and terminal deoxynucleotidyl-transferase-mediated dUTP nick-end labeling (TUNEL) assay kit were purchased from Thermo Scientific (Thermo Scientific, Cat# C10617, Rockford, IL).

### Animals

All animal studies were approved by the University of Pittsburgh Medical Center Institutional Animal Care and Use Committee, which adhere to the National Institutes of Health Guide for the Care and Use of Laboratory Animals, and reported in accordance with the Animal Research: Reporting In Vivo Experiments (ARRIVE) guidelines 2.0 [24]. The C57Bl/6J strain mice (male and female, 2–3 months old) used in the study were purchased from Jackson laboratories (Bar Harbor, ME). Mice were housed in a temperature-controlled room on a 12-h light/12-h dark cycle with standard mouse diet and water ad libitum.

### Ischemic stroke model

Ischemic stroke was induced by transient middle cerebral artery occlusion (tMCAO) and reperfusion as previously described [18]. Briefly, mice were anesthetized with 3% isoflurane vaporized in N_2_O and O_2_ (3:2) for induction and 1.5% isoflurane for maintenance. The left common carotid artery (CCA) was exposed through a midline pre-tracheal incision under an operating microscope. The CCA were isolated and ligated. The external carotid artery (ECA) was dissected further distally and permanently ligated. The internal carotid artery (ICA) was isolated and carefully separated from the adjacent vagus nerve. To occlude the left middle cerebral artery (MCA), a rubber silicon-coated monofilament suture (size 6–0, diameter with coating 0.21 ± 0.02 mm; coating length 5–6 mm) was introduced into the ECA and advanced along the ICA 8–9 mm from the bifurcation of the carotid artery and blocked MCA flow for 60 min. The silk suture around the ECA stump was tightened around the intraluminal rubber suture to prevent bleeding. For reperfusion (Rp), the suture was gently withdrawn to restore blood flow. The sham surgery mice received the same operation, but the monofilament suture was not inserted. Body temperature was maintained for the duration of the experiment between 36.5 ± 0.5 °C with a small-animal heating pad. Post-surgery, softened mouse food and water were provided to accelerate recovery.

### Drug treatment

Post-stroke mice were randomly assigned to receive either vehicle (Veh, 100% DMSO, 2 ml/kg body weight/day), novel SPAK inhibitor ZT-1a (5 mg/kg body weight/day) or NKCC1 inhibitor bumetanide (BMT, 10 mg/kg body weight/day) via intraperitoneal injection (i.p.), with an initial half-dose administered at 3 h and the second half-dose at 8 h Rp as shown in Fig. 2a.

### Immunofluorescent staining and image analysis

Mice were anesthetized with 3% isoflurane and transcardially perfused with ice-cold normal saline, followed by ice-cold 4% paraformaldehyde (PFA) in 0.1 M PBS as described before [25]. Brains were dissected and kept in 4% PFA for 24 h before being transferred to a 30% sucrose solution for cryoprotection. Coronal brain sections (25 µm thick, at + 0.62 to − 0.82 mm posterior from Bregma) were selected and incubated with blocking solution (10% normal goat serum (NGS) + 0.5% Triton X-100 for 1 h at room temperature (RT)) followed by incubation with primary antibodies (Additional file 1: Table S1) in the blocking solution (3% NGS and 0.3% Triton X-100 in PBS) for overnight at 4 °C. On the following day, brain sections were washed three times in PBS and then incubated for 1 h at RT with respective secondary antibodies: goat anti-rabbit Alexa 488-conjugated IgG (1:200), goat anti-rabbit Alexa 546-conjugated IgG (1:200), goat anti-mouse Alexa 546-conjugated IgG (1:200), goat anti-rat Alexa 546 (1:200). After washing for 3 × 10 min, nuclei were stained with DAPI (1:1000, in the blocking solution) for 15 min at RT. Sections were mounted with Vectashield mounting medium (Vector Laboratories, Burlingame, CA). For negative controls, brain sections were stained with the secondary antibodies only (Additional file 1: Fig. S1). Fluorescent images were captured with Olympus IX81 inverted microscope with a FV1000 laser scanning confocal system using a 40 × oil-immersion objective. Identical digital imaging acquisition parameters were used and analyzed by a blinded observer throughout the study. Fluorescence images were quantified with Fiji (NIH) software.

For CPEC cultures, CPECs grown on coverslips were fixed in 4% PFA in PBS for 15 min. After rinsing with PBS, cells were incubated with a blocking solution (10% normal horse serum (NHS), 2% BSA and 0.25% Triton X-100 in 0.1 M PBS) for 1 h at RT followed by incubation with primary antibodies (Additional file 1: Table S1) overnight at 4 °C. After rinsing in PBS, slides were incubated with appropriate goat or mouse Alexa fluor 546/488 secondary antibodies (1:200, Invitrogen) for 1 h at RT. Fluorescence images were captured as described above.

### Brain infarct volume measurement

Loss of microtubule-associated protein 2 (MAP2) as a marker for neurodegeneration was analyzed for infarct volumetric assessment as described before [25]. Brain sections at different levels (0.62, 0.14, − 0.34, − 0.82 mm posterior from Bregma) from each brain were selected. Fluorescent images of MAP2 were captured using a 4 × objective with a Nikon Eclipse Ti epifluorescent microscope (Nikon, Tokyo, Japan) and processed with NIS-Elements Advance Research microscope imaging software (version 4.30.02, Nikon). The ischemic area for each brain slice was calculated by subtracting the non-infarct area in the ipsilateral (IL) hemisphere from the total area of the contralateral (CL) hemisphere with Image J.

### Immunoblotting

ChP tissue or cultured cells were harvested and incubated in RIPA buffer containing one pill of phosSTOP and 2 mM protease inhibitors as described before [26]. Protein concentration was measured with the bicinchoninic acid (BCA) assay kit by using a 96-well microplate reader (Spectra Max 190; Molecular Devices). Protein samples (30 µg cellular lysates, or 7 µg ChP tissue homogenates) were boiled in sample buffer (Thermo Scientific, Rockford, IL) for 7 min, resolved by 4–15% sodium dodecyl sulfate polyacrylamide-gel electrophoresis and electrotransferred onto a polyvinylidene difluoride (PVDF) membranes [19, 27]. The membranes were blocked in 5% BSA or 7.5% nonfat dry milk in Tris-buffered saline-T (TBS-T, 0.05% Tween-20) for 1 h at RT and then incubated with appropriate primary antibodies (Additional file 1: Table S1) overnight at 4 °C. Blots were then washed six times with TBS-T and incubated with secondary horseradish peroxidase-conjugated antibodies (1:1000 or 1:2000) in 5% nonfat dry milk in TBS-T for 1 h at RT. Protein bands were visualized with ECL agents. The densities of protein bands were analyzed with Image J. Expression of GAPDH or α-tubulin was used as a protein loading control (Raw Immunoblot Images are shown).

### ChP sample preparation and flow cytometric analysis

Mice were euthanized with CO2 and transcardially perfused with ice-cold PBS [25]. Lateral ventricle choroid plexus (LVCP) were carefully isolated under a microscope and placed in 1.5-ml tubes with ice-cold PBS. CL or IL ChP were enzymatically digested in collagenase type IV (400 U/ml in PBS, 45 min, 37 °C), and then triturated several times using a pipette [28]. Cells were stained with antibodies (Additional file 1: Table S1) for 20 min at 4 °C in the dark. Samples were acquired using an LSRFortessa flow cytometer (BD Biosciences, USA) equipped with FACS Diva software and a minimum of 30,000 events for each sample was recorded. Data were analyzed using the Flow Jo (BD Biosciences, USA) software.

### Primary cultures of CPECs

C57Bl/6J mice (2–3 months old) were deeply anesthetized with 3% isoflurane and transcardially perfused with ice-cold PBS (without Ca^2+^ and Mg^2+^). The brain was removed and immediately submerged in the ice-cold PBS. The ChP tissues (both lateral ventricles, third ventricle and fourth ventricle) were dissected out under a microscope and washed with ice-cold HBSS. The ChP tissues were incubated with trypsin (0.25 mg/ml) at 37 °C for 20 min and cells were dissociated by pipetting. The cell suspension was washed in culture medium (DMEM/F12) supplemented with 10% heat-inactivated FBS, 1 mM l-glutamine, 1 mM sodium pyruvate, 100 U/ml penicillin, 100 mg/ml streptomycin, 5 μg/ml insulin, 20 μM Ara-C, 5 ng/ml sodium selenite, and 10 ng/ml human EGF as described before [29]. The cells were plated on glass coverslips (coated with poly-l-lysine, 24-well plate, ~ 2 × 10^5^ cells/well) and cultured at 37 °C, 5% CO_2_, and refed with the fresh media on the following day. CPEC cultures for 5–6 days in vitro (DIV) were used in this study.

### Primary cortical neuron cultures

Embryonic day 14–18 pregnant mice (C57Bl/6J background) were euthanized with CO_2_ with a secondary method of euthanasia by cervical dislocation as described previously [30]. Fetuses were removed and decapitated. Using a dissection microscope, the cortices were extracted from the brain while submerged in ice-cold HBSS and treated with 0.125 mg/ml trypsin at 37 °C for 20 min. Once the trypsin was neutralized with normal DMEM, the cells were centrifuged at 1200 RPM for 5 min at 20 °C. The cells were washed once in normal DMEM and centrifuged. The cells were then suspended in neurobasal medium containing B-27 supplement (2%), 100 U/ml GlutaMAX and 0.1 mg/ml penicillin/ streptomycin. The cells were seeded in 6/24-well plates (5 × 10^5^ or 1.5 × 10^5^ cells/well) or on glass coverslips (coated with PDL) and incubated at 37 °C in an incubator with 5% CO_2_ and atmospheric air. Neuronal cultures 7 to 10 DIV were used in this study.

### In vitro oxidative stress induction and drug treatment

Cultured CPECs in 24-well plates were treated with either the normal medium, H_2_O_2_ (200 µM), H_2_O_2_ (200 µM) + Ebselen (1 µM), H_2_O_2_ (200 µM) + ZT-1a (1 µM), or H_2_O_2_ (200 µM) + BMT (10 µM) for 24 h. For cultured neurons, cells were treated with either the normal medium, H_2_O_2_ (20 µM), H_2_O_2_ (20 µM) + Ebselen (100 nM), H_2_O_2_ (20 µM) + ZT-1a (1 µM), or H_2_O_2_ (20 µM) + BMT (10 µM) for 24 h. Cultures were collected for imaging or immunoblot experiments.

### TUNEL assay of cultured CPECs

The CPEC cultures were fixed in 4% PFA in PBS for 15 min. Oxidative stress-induced apoptotic cell death was detected by TUNEL staining according to the manufacturer’s instructions. The double-labeled cells were imaged with Olympus IX81 inverted microscope with a FV1000 laser scanning confocal system under 40 × objective. Four independent areas were quantified from each coverslip and apoptotic cell death rate in each experiment was expressed as the ratio of TUNEL^+^/Cytokeratin^+^ cells and Cytokeratin^+^/DAPI^+^ cells.

### Measurement of neuronal survival

Cell viability of cultured neurons was assessed by retention of Calcein-AM (live) and propidium iodide (PI) uptake (damaged) cells as described previously [31]. Under the Nikon TiE inverted epifluorescence microscope, at least four independent areas at 20 × magnification were quantified from each coverslip with ImageJ [18]. Cell viability was expressed as the ratio of calcein^+^ neurons to the sum of calcein^+^ and PI^+^ neurons.

### Cellular rubidium (Rb^+^) influx and intracellular Na^+^ concentration measurement

After H_2_O_2_ ± drug treatment, neuronal cells were rinsed with an isotonic Rb^+^-free wash buffer (310 mOsm, containing 134 mM NaCl, 2 mM CaCl_2_, 0.8 mM NaH_2_PO_4_, 5 mM glucose, 25 mM HEPES and 1.66 mM MgSO_4,_ PH 7.4) as described before [26]. Cells were exposed to isotonic buffer (310 mOsm, containing 5.36 mM Rb^+^) solutions with or without BMT (10 µM) for 10 min at 37 °C. To terminate Rb^+^ influx, cells were washed with wash buffer (Rb^+^ free) and lysed with 0.15% SDS (200 μl/well) to release the intracellular Rb^+^. The intracellular Rb^+^ concentration in cell lysates was measured using an automated atomic absorption spectrophotometer (Ion Channel Reader, ICR-8000; Aurora Biomed, Vancouver, Canada). Total protein of cell lysates was measured by BCA assay. NKCC1-mediated Rb^+^ influx assay was determined by subtracting Rb^+^ influx value in the presence of BMT from one in the absence of the BMT. Rb^+^ influx rate was calculated and presented as μg Rb^+^/mg protein/min.

Intracellular Na^+^ concentration ([Na^+^]_i_) was measured with the fluorescent dye SBFI/AM as described previously with some modifications [32]. Cells grown on coverslips were loaded with 30 µM SBFI/AM added with 0.02% pluronic acid. The coverslips were placed in the open bath imaging chamber and incubated with HEPES-MEM buffer at 37 °C. The Nikon Ti Eclipse inverted epifluorescence microscope and a × 40 oil-immersion lens were used. The samples were excited at 340 and 380 nm and the ratios of 340/380 were analyzed with the MetaFluor image-processing software. Absolute [Na^+^] was detected for each cell by performing an in situ calibration as described previously [33].

### Statistical analyses

A total of 130 C57Bl/6J male and female mice were used in this study. All data assessments were performed by an investigator who was blind to treatment groups and/or experimental group assignments. Student’s two-tailed *t*-test was used to compare between two experimental groups. For three or more groups, one-way ANOVA was conducted for multiple comparisons. *N* values represent the number of independent experiments. Data were expressed as mean and standard deviation (SD). A *p* < 0.05 was considered statistically significant. Data were graphed using GraphPad Prism 8 (GraphPad Software, Inc., CA, USA).

## Results

### Ischemic stroke triggered phosphorylated activation of the SPAK–NKCC1 cascade pathway in the ChP

**Fig. 1:**
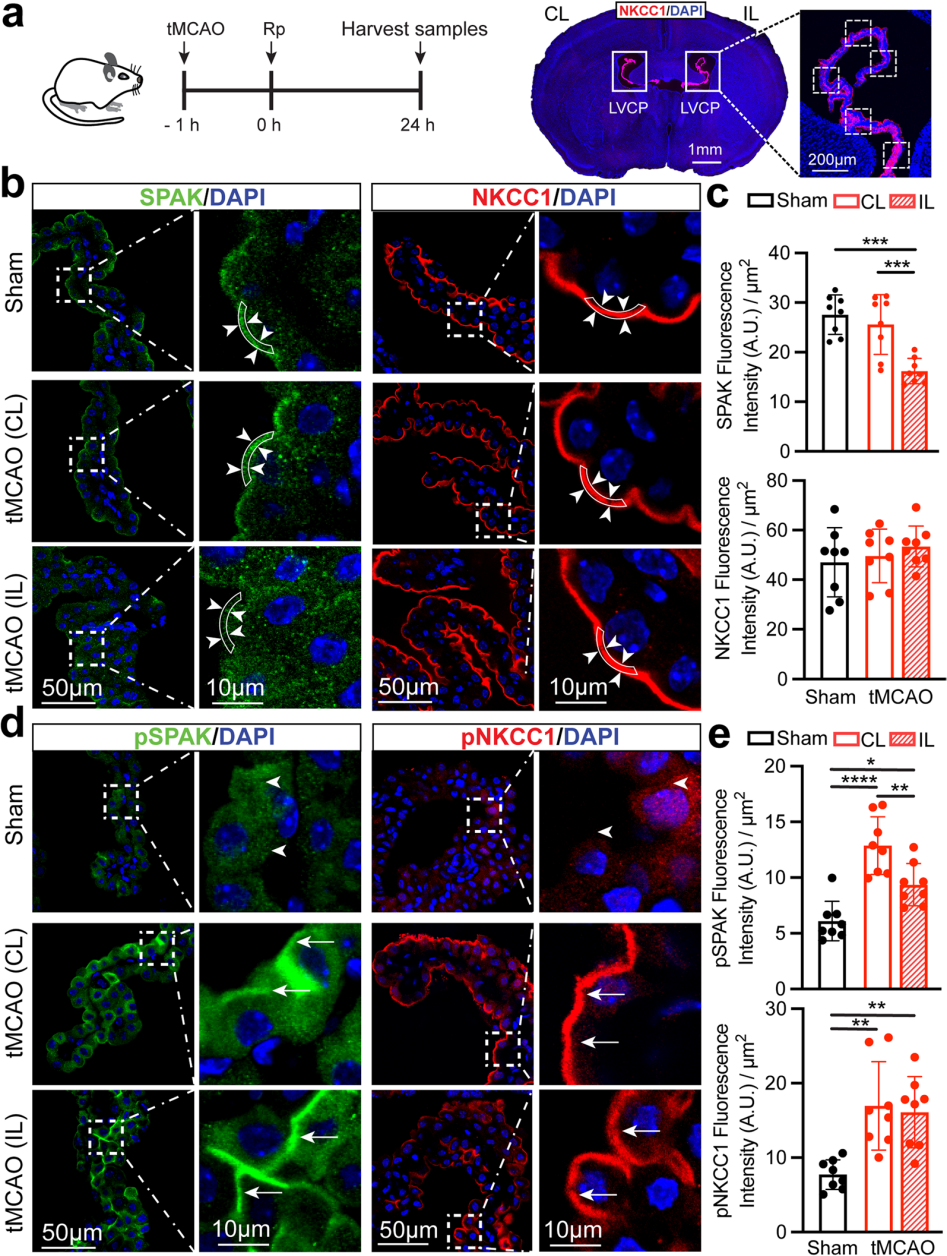
Stroke-induced phosphorylated activation of SPAK–NKCC1 complex in the choroid plexus. **a** Experimental protocol. Changes of SPAK–NKCC1 protein complex in the lateral ventricle choroid plexus (LVCP) from Sham or tMCAO mice were assessed by immunostaining, as illustrated in representative images of contralateral (CL) and ipsilateral (IL) LVCP stained for the NKCC1 protein. **b** Representative confocal fluorescence images of SPAK and NKCC1 expression. Arrowheads: apical membrane (region of interest) expression of SPAK or NKCC1 protein. **c** Quantification summary. Data are mean ± SD (*n* = 8). ***p < 0.001, ****p < 0.0001. One-way ANOVA. **d** Representative confocal fluorescence images of the phosphorylated species (active) of pSPAK or pNKCC1 protein. Arrowheads: low level of pSPAK and pNKCC1 in Sham groups. Arrows: elevated pSPAK or pNKCC1 expression in the post-stroke ChP. **e** Quantification summary. Data are mean ± SD (*n* = 8). *p < 0.05, **p < 0.01, ****p < 0.0001. One-way ANOVA

We assessed changes of SPAK–NKCC1 complex expression in the lateral ventricle choroid plexus (LVCP) in Sham or stroke brains at 24 h after Rp (Fig. 1a), a peak time for stroke-induced ChP damage [34]. In the Sham brains, abundant SPAK and NKCC1 proteins were detected in the apical (luminal) membranes of CPECs (region of interest, ROI, Fig. 1b), but rarely in the basolateral membrane, consistent with previous literature reports [12, 35]. Compared to Sham, ischemic stroke triggered a significant reduction of SPAK protein expression in the ipsilateral LVCP (ROI, Fig. 1b and c, *p* < 0.001) but did not significantly alter the expression of SPAK or NKCC1 proteins in the non-stroke contralateral (CL) LVCP (Fig. 1b and c, *p* > 0.05). In the case of the phosphorylated protein species SPAK (pSPAK, Ser373) and NKCC1 (pNKCC1, Thr212/Thr217), Sham brains showed low expression of both proteins in CPECs, as reported before [7]. In contrast, ischemic stroke triggered upregulation of pSPAK and pNKCC1 expression at the apical membranes of CPECs in both hemispheres of stroke brains (arrows, Fig. 1d and e, *p* < 0.05). Elevation of pNKCC1 was further validated with a different antibody (pT58 NCC, which cross-reacts with the NKCC1 pThr211 phosphorylation site, Additional file 2: Fig. S2a). These findings demonstrated that ischemic stroke altered SPAK–NKCC1 complex at the ChP by increasing their phosphorylatory stimulation as well as increasing the polarized protein trafficking to the apical membrane. These changes may subsequently impact the ChP blood–CSF barrier structures and function.

### Pharmacological inhibition of SPAK–NKCC1 cascade reduced stroke-induced infarct volume and ChP damage

**Fig. 2:**
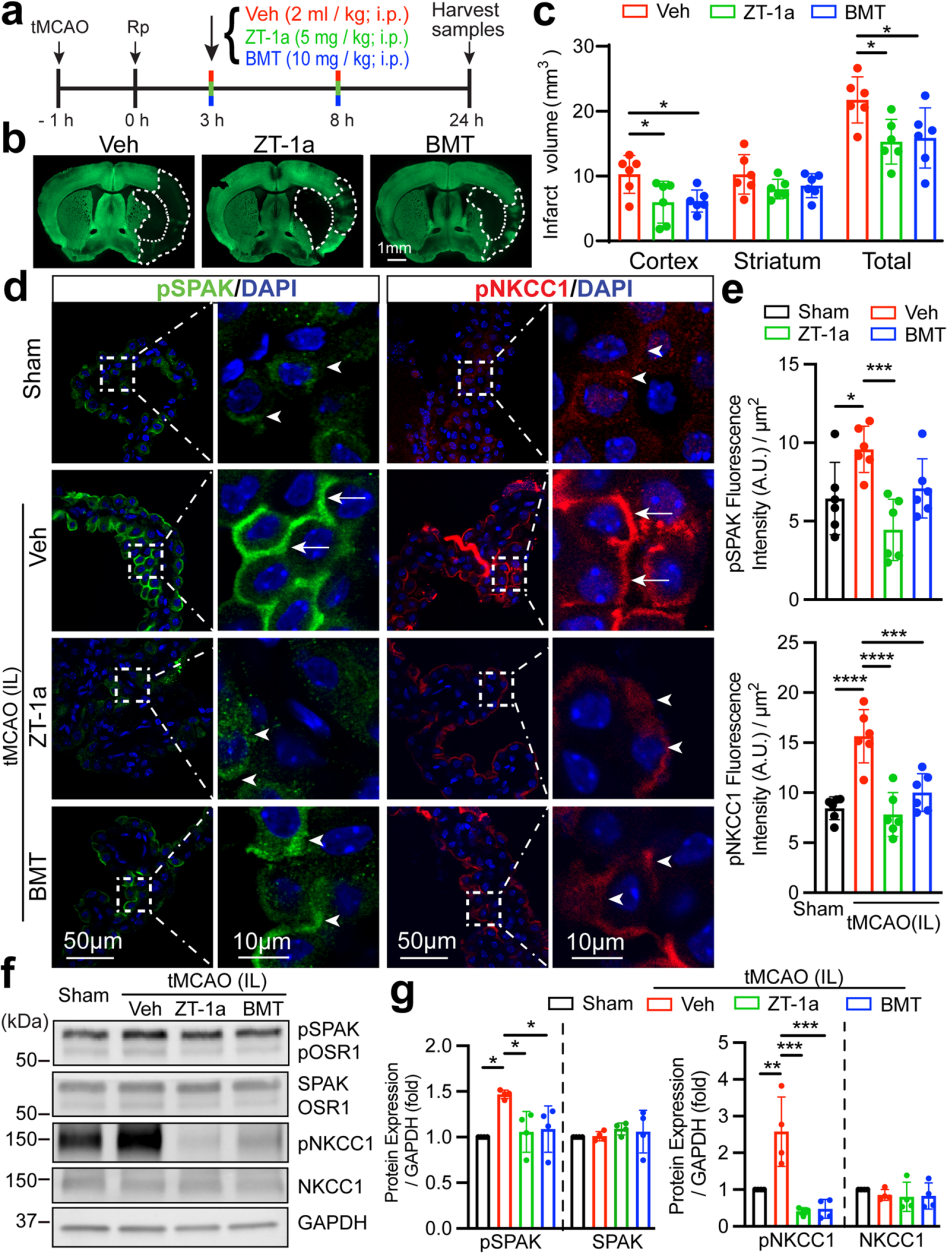
Pharmacological suppression of SPAK–NKCC1 complex phosphorylation in choroid plexus after ischemic stroke. **a** Experimental protocol. Mice were subjected to tMCAO and randomly assigned to vehicle (Veh), ZT-1a or bumetanide (BMT) treatment group administered via intraperitoneal injection (i.p.) with a half-dose at 3 h and 8 h post-reperfusion (Rp), respectively. **b** Representative infarct images with MAP2 expression staining at 24 Rp. **c** Infarct volume quantification summary. Data are mean ± SD (*n* = 6, 4 male, 2 female). *p < 0.05. One-way ANOVA. **d** Representative immunofluorescent images of pSPAK and pNKCC1 staining in the Sham or ipsilateral (IL) LVCP post-stroke brains. Arrowheads: low level expression of pSPAK or pNKCC1. Arrows: elevated pSPAK or pNKCC1 expression. **e** Quantification summary. Data are mean ± SD (*n* = 6, 4 male, 2 female). *p < 0.05, ***p < 0.001, ****p < 0.0001. One-way ANOVA. **f** Western blot analysis of SPAK–NKCC1 cascade expression in the ChP of Sham, stroke Veh-control, ZT-1a- or BMT-treated mice. ChP tissue lysates were subjected to immunoblotting with the indicated antibodies. **g** Immunoblot summary. Data are mean ± SD (*n* = 4, 2 male, 2 female). *p < 0.05, **p < 0.01, ***p < 0.001. One-way ANOVA

Our recent study shows that pharmacological inhibition of SPAK protein activity with its inhibitor ZT-1a attenuated ischemic stroke brain injury or ChP CSF hypersecretion in experimental hemorrhagic hydrocephalus [19]. In addition, inhibition of the ChP NKCC1 with its inhibitor bumetanide (BMT) reduced traumatic brain injury (TBI)-induced brain edema, cerebral contusion, and neuronal death [36]. Figure 2a–c demonstrates that, in comparison to the Veh-treated stroke control group, systematic administration of SPAK inhibitor ZT-1a or NKCC1 inhibitor BMT significantly decreased total infarct volume (~ 30% or 27%, respectively, *p* < 0.05), and cortical infarct volume by ~ 42% or 40%, respectively (*p* < 0.05). We then examined the efficacy of ZT-1a or BMT on decreasing stroke-induced damage of ChP. As shown in Fig. 2d and e, in the Veh-treated stroke control brains, expression of pSPAK and pNKCC1 at the IL LVCP was significantly elevated (arrows, *p* < 0.05). In contrast, ZT-1a, but not BMT, decreased the expression of pSPAK protein by ~ 54% (arrowhead, Fig. 2d and e, top panel, *p* < 0.001). ZT-1a or BMT treatment also significantly reduced expression of pNKCC1 in the IL LVCP by ~ 50% or ~ 36%, respectively (Fig. 2d and e, bottom panel, *p* < 0.001). Consistent with our immunofluorescent staining, quantification of pSPAK and pNKCC1 expression via immunoblotting analysis clearly shows that stroke-induced elevation of pSPAK and pNKCC1 expression in the ChP was blocked by ZT-1a or BMT treatment (Fig. 2f and g, *p* < 0.05). Similar patterns of changes were detected in CL LVCP (Additional file 2: Fig. S2b–e). Moreover, compared to Sham, ischemic stroke triggered a significant reduction of SPAK protein expression in the ipsilateral LVCP (ROI, Additional file 1: Fig. S3a and b, *p* < 0.01). Both drug treatments restored the SPAK protein expression in the apical membrane of IL LVCP (ROI, Additional file 1: Fig. S3a and b). However, no significant changes of SPAK or NKCC1 expression among the three groups were detected via immunoblotting (Fig. 2f and g, *p* > 0.05). These findings clearly demonstrate that pharmacological blockade of the SPAK–NKCC1 complex prevented stroke-induced phosphorylatory activation of the complex in the ChP.

### Pharmacological inhibition of SPAK–NKCC1 signaling complex preserved ChP blood–CSF barrier integrity

**Fig. 3:**
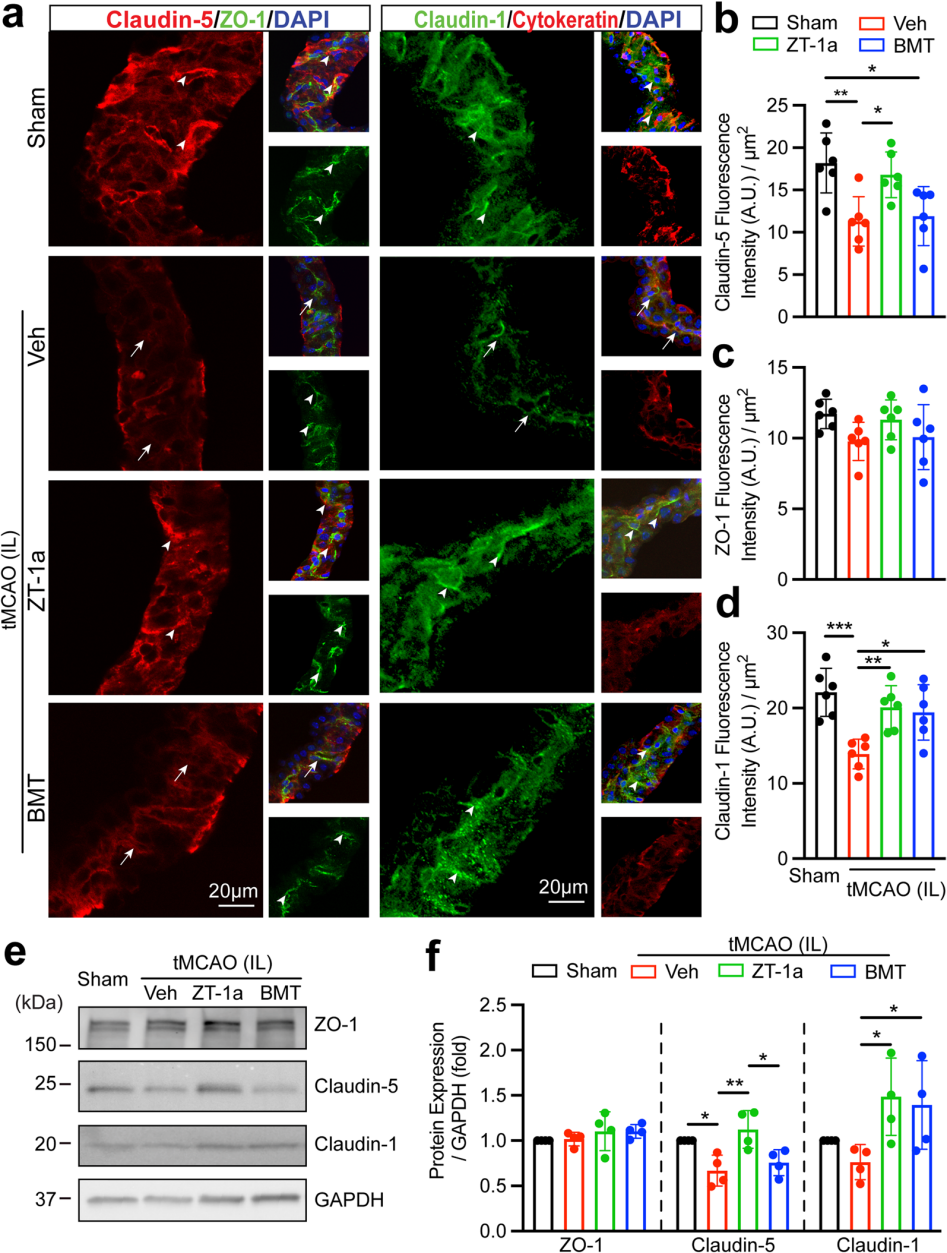
Pharmacological inhibition of SPAK–NKCC1 signaling complex increased choroid plexus tight junction integrity after ischemic stroke. **a** Representative confocal images of tight junction proteins (Claudin-5, ZO-1, and Claudin-1) as well as the epithelial marker cytokeratin in the IL LVCP in Sham, stroke Veh-control, ZT-1a- or BMT-treated mice at 24 h Rp. Arrowheads: expression of Claudin-5, ZO-1 and Claudin-1. Arrows: Low level expression of Claudin-5 or Claudin-1. **b–d** Summary data of Claudin-5, ZO-1 and Claudin-1 fluorescence intensity. Data are mean ± SD (*n* = 6, 4 male, 2 female). *p < 0.05, **p < 0.01, ***p < 0.001. One-way ANOVA. **e** Western blot analysis of ZO-1, Claudin-5 and Claudin-1 TJ protein expression in the IL LVCP of Sham, stroke Veh-control, ZT-1a- and BMT-treated mice at 24 h Rp. ChP tissue lysates were subjected to immunoblotting with the indicated antibodies. **f** Immunoblot quantitation. Data are means ± SD (*n* = 4, 2 male, 2 female). *p < 0.05, **p < 0.01. One-way ANOVA

**Fig. 4:**
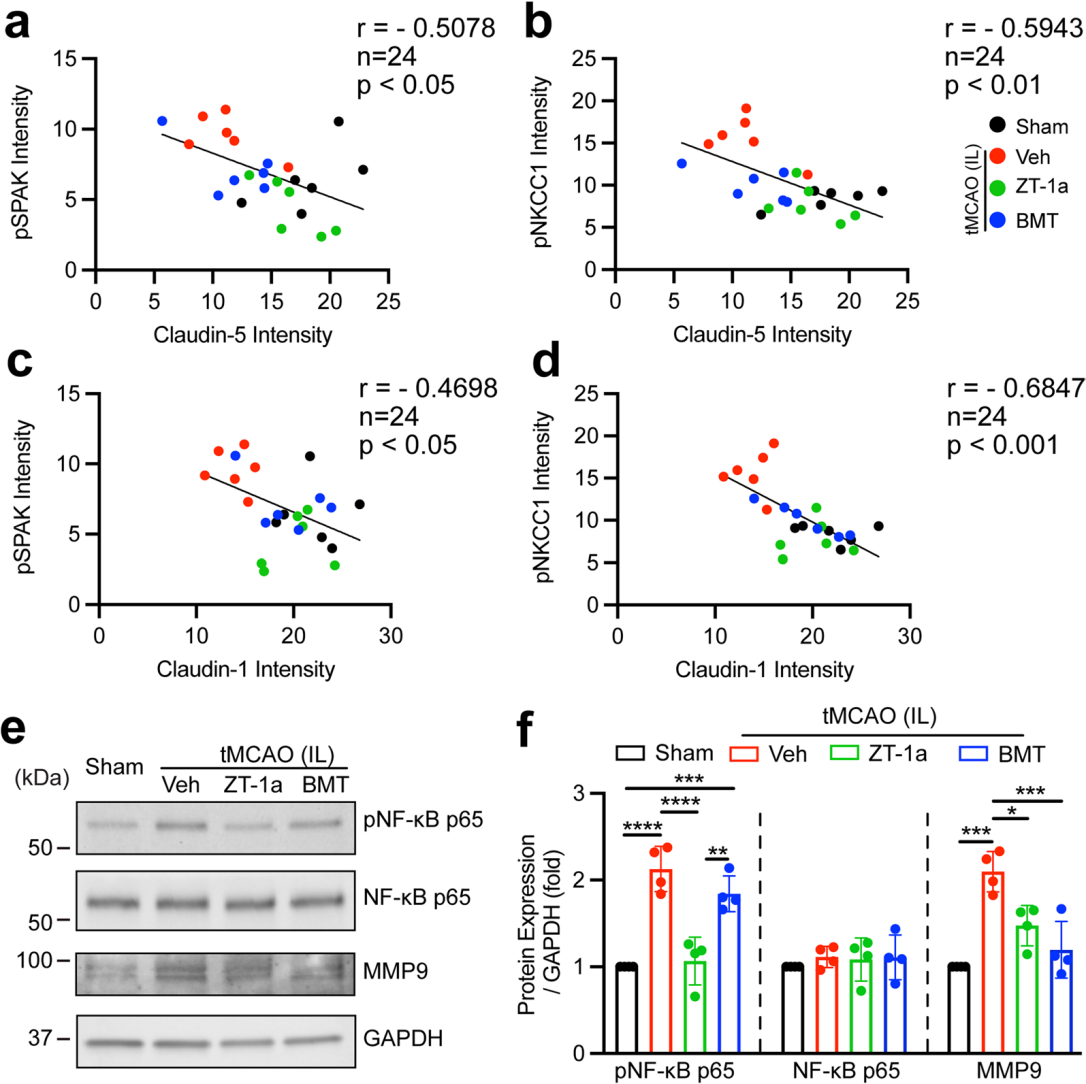
Changes of SPAK–NKCC1 signaling complex and NF-kB activation in the ChP after stroke. **a** and **c** Pearson correlation analysis between pSPAK and Claudin-5 or Claudin-1 fluorescence intensity in the IL ChP (from the data presented in Fig. 3a–d). **b** and **d** Pearson correlation between pNKCC1 and Claudin-5 or Claudin-1 fluorescence intensity in the IL ChP. *n* = 24. **e** Western blot analysis of pNF-κB p65, NF-κB p65, and MMP9 protein expression in the IL LVCP of Sham, stroke Veh-control, ZT-1a- or BMT-treated mice at 24 h Rp. ChP tissue lysates were subjected to immunoblotting with the indicated antibodies. **f** Immunoblot quantitation. Data are mean ± SD (*n* = 4, 2 male, 2 female). *p < 0.05, ***p < 0.001, ****p < 0.0001. One-way ANOVA

We subsequently investigated whether pharmacological inhibition of SPAK–NKCC1 signaling complex attenuates stroke-induced ChP blood–CSF barrier dysfunction. Downregulation of TJ Claudin-5, Claudin-1 and ZO-1 gene in the ChP tissues is associated with increased blood–CSF barrier permeability in AD mouse model [37]. In addition, reduced Claudin-5 gene and protein in the ChP was also detected in AD patients [38] and Huntington’s disease patients [39]. We examined whether upregulation of SPAK–NKCC1 signaling pathway in the CPECs is associated with changes in the TJ integrity and whether pharmacological inhibition of SPAK–NKCC1 complex attenuates stroke-induced ChP blood–CSF barrier dysfunction in ischemic brains. As shown in Fig. 3a (left panel), the reduced Claudin-5 protein expression was detected in the stroke Veh-control ChP (Fig. 3a and b, *p* < 0.01). In comparison to the Veh stroke brains, ZT-1a-treated stroke brains, but not the BMT-treated, exhibited statistically significant preservation of Claudin-5 protein expression in the ChP (Fig. 3a and b, *p* < 0.05). We also assessed changes of Claudin-1 expression, which plays important role in ChP development and blood–CSF barrier function [40]. Figure 3a (right panel) displays abundant Claudin-1 expression in the Sham brain CPECs. The Veh-treated stroke mice exhibited loss of Claudin-1 protein expression in IL LVCP. But pharmacological inhibition of the SPAK–NKCC1 complex prevented down-regulation of Claudin-1 expression (Fig. 3a and d). However, ischemic stroke did not cause detectable changes of ChP ZO-1 protein expression in all groups (Fig. 3a and c). We further validated these findings via immunoblotting of ChP under the above conditions. Compared to Sham, stroke brains displayed pronounced reduction of Claudin-5 protein expression in the IL LVCP (Fig. 3e and f, *p* < 0.05). Pharmacological inhibition of SPAK–NKCC1 signaling complex with ZT-1a but not BMT exhibited preservation of Claudin-5 protein expression in the ChP (Fig. 3e and f, *p* < 0.01). Both inhibitors are effective in maintaining Claudin-1 protein expression in the ChP (Fig. 3e and f, *p* < 0.05). There are no changes of ZO-1 protein expression (Fig. 3e and f, *p* > 0.05). Moreover, CL LVCP exhibited a similar pattern of changes under above conditions (Additional file 1: Fig. S5a—d). Lastly, Pearson’s correlation analysis revealed that the fluorescence intensity of ChP pSPAK protein expression was negatively correlated with Claudin-5 (*r* = − 0.5078, Fig. 4a, *p* < 0.05) or with Claudin-1 expression fluorescence intensity (*r* = − 0.4698, Fig. 4c, *p* < 0.05). The pNKCC1 protein expression also showed negative correlations with Claudin-5 and Claudin-1 protein expression (*r* = − 0.5943, *r* = − 0.6847, respectively, Fig. 4b and d, *p* < 0.01). Consistently, no clear correlation between changes of SPAK–NKCC1 signaling cascade and ZO-1 protein expression in all groups (*r* = − 0.1752, *r* = − 0.2976, respectively, Additional file 1: Fig. S4, *p* > 0.05). In summary, these findings suggest that activation of SPAK–NKCC1 signaling cascade pathway is closely associated with TJ damage in the ChP after ischemic stroke.

### Stroke-mediated activation of NF-kB signaling in ChP

It was reported that pro-inflammatory cytokine-induced activation of matrix metalloproteinases (MMPs) and reactive oxygen species (ROS) mediated stimulation of the NF-κB signaling are involved in TJ degradation at the BBB and vasogenic edema in stroke brains [41, 42]. We then determined whether these signaling mechanisms play a role in stroke-mediated damage at the blood–CSF barrier. The ChP isolated from the Veh-treated stroke brains showed upregulated expression of the activated NF-κB (pNF-κB p65) by ~ 50%, compared to the Sham ChP (Fig. 4e and f, *p* < 0.0001). There were no changes in the total NF-κB protein expression (Fig. 4e and f, *p* > 0.05). Interestingly, SPAK inhibitor ZT-1a treatment, but not NKCC1 inhibitor BMT, blocked NF-κB activation (Fig. 4e and f, *p* < 0.0001). Moreover, compared to the Sham ChP, a significant increase in the expression of MMP9 protein was detected in the Veh-treated stroke ChP (Fig. 4e and f, *p* < 0.001). Pharmacological inhibition of SPAK–NKCC1 complex with ZT-1a or BMT reduced MMP9 protein expression (~ 32% or by ~ 42%, respectively, Fig. 4e and f, *p* < 0.05). Moreover, CL LVCP exhibited similar changes under above conditions (Additional file 1: Fig. S5e and f). Together, this data suggests that NF-κB and MMP9 signaling pathways are activated in ChP after stroke and play a role in damaging the blood–CSF barrier. Blocking SPAK–NKCC1 complex attenuated NF-κB and MMP9 signaling activation and ChP TJ degradation.

### Pharmacological inhibition of SPAK–NKCC1 complex reduced the ChP blood–CSF barrier permeability after stroke

To investigate impact of blocking SPAK–NKCC1 signaling cascade on the ChP blood–CSF barrier permeability in these brains, we evaluated immunofluorescence staining of endogenous albumin distribution at the ChP. As shown in Additional file 1: Fig. S6a, Sham ChP displayed localization of endogenous albumin protein restricted to the ChP fenestrated vessels (stained with endothelial marker glucose transporter 1, GLUT 1), consistent with other reports on low permeability of macromolecules, such as albumin, at the ChP blood–CSF barrier in adult brains [43]. In contrast, stroke brains showed altered albumin distribution in the IL LVCP, reflected with loss of vasculature albumin (leak out from ChP), and/or increased albumin uptake in the CPECs (Additional file 1: Fig. S6a and b, *p* < 0.01). Interestingly, ZT-1a-treated stroke brains exhibited restored retention of albumin immunoreactive signals in the ChP vessels, as seen in Sham brains, and less in the CPECs (Additional file 1: Fig. S6a and b). These findings illustrate that the ChP blood–CSF barrier permeability was compromised after ischemic stroke, and pharmacological blockade of SPAK–NKCC1 complex protected the ChP blood–CSF barrier integrity.

### Pharmacological inhibition of SPAK–NKCC1 complex reduced immune cell infiltration in the ChP after stroke

**Fig. 5:**
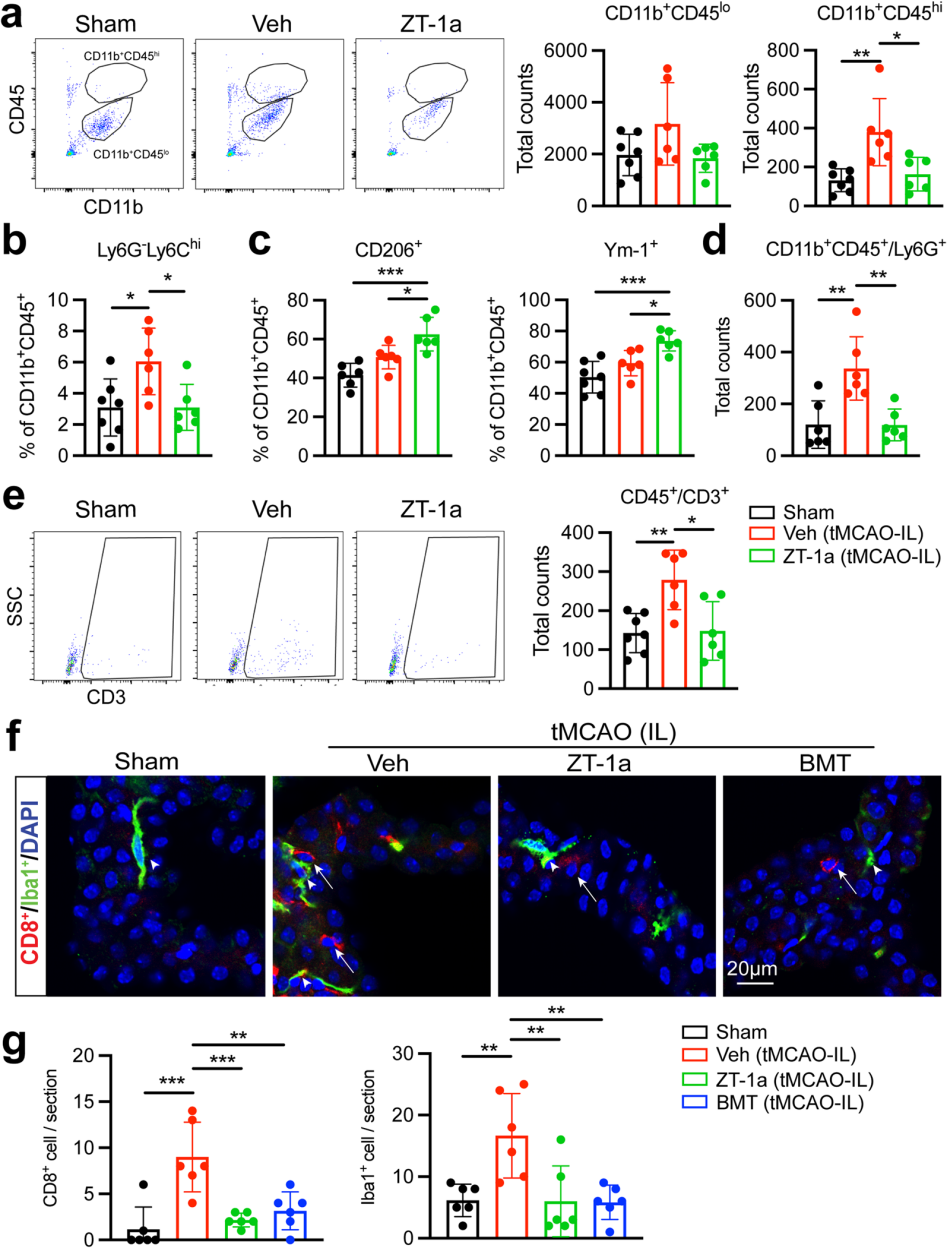
Pharmacological inhibition of SPAK–NKCC1 signaling reduced infiltration of immune cell in the ChP. **a** Representative flow cytometric plots of CD11b^+^CD45^lo^ or CD11b^+^CD45^hi^ myeloid cells from the isolated IL ChP at 3 days post-surgery with quantification of total number of CD11b^+^CD45^lo^ or CD11b^+^CD45^hi^ myeloid cells in the ChP. **b** and **c** Percentage of CD11b^+^CD45^+^Ly6G^−^Ly6C^hi^ and CD206^+^ and Ym-1^+^ cells gated within CD11b^+^CD45^+^ cells. **d** Total number of CD11b^+^CD45^+^Ly6G^+^ neutrophils in the ChP. **e** Representative flow cytometric plots and the total number of CD3^+^ T cells in the ChP. Data are mean ± SD (*n* = 6–7). *p < 0.05, **p < 0.01. One-way ANOVA. **f** Representative images of CD8^+^ T cells (arrows) and Iba1^+^ microglia cells (arrowheads) in the IL LVCP in Sham, stroke Veh-control, ZT-1a- or BMT-treated stroke mice at 24 h Rp. **g** Quantification of CD8^+^ T cells and Iba1^+^ microglia cells per one coronal section in the IL hemispheres. Data are mean ± SD (*n* = 6, 4 male, 2 female). **p < 0.01, ***p < 0.001. One-way ANOVA

ChP regulates immune cells infiltration to the CNS [28] and is a route for lymphocyte invasion into the brain after acute ischemic stroke and neuroinflammatory responses [11]. We next assessed whether pharmacological inhibition of SPAK–NKCC1 cascade has an impact on circulating leucocyte infiltration in the ChP. With the flow cytometric analysis of the immune cells in the isolated ChP at day 3 post-stroke, CD11b^+^CD45^lo^ myeloid cells were unchanged between Sham, Veh-treated or ZT-1a-treated ChP, while the number of CD11b^+^CD45^hi^ myeloid cells increased by 65% in the Veh-treated ChP, compared to the Sham ChP (Fig. 5a, *p* < 0.01). ZT-1a treatment reduced the number of CD11b^+^CD45^hi^ cells by 50% (Fig. 5a, *p* < 0.05). CD11b^+^CD45^+^Ly6G^−^Ly6C^hi^ inflammatory cells were significantly increased in the Veh-control ChP (Fig. 5b, *p* < 0.05). However, ZT-1a-treated mice displayed reduced infiltration of CD11b^+^CD45^+^Ly6G^−^Ly6C^hi^ inflammatory cells in the ChP (Fig. 5b, *p* < 0.05). In addition, the CD206^+^ and Ym-1^+^ anti-inflammatory myeloid cells were increased by 20% in the ZT-1a-treated ChP, compared to the Veh-control ChP (Fig. 5c, *p* < 0.05). A 2.8-fold increase of CD11b^+^CD45^+^/Ly6G^+^ neutrophils and a twofold increase in CD3^+^ T cells were detected in the IL ChP of the Veh-treated stroke mice, compared to the Sham ChP (Fig. 5d and e, *p* < 0.01). ZT-1a-treated mice displayed reduced infiltration of neutrophils and CD3^+^ T cells by 65% and 47%, respectively (Fig. 5d and e, *p* < 0.05). Similar flow cytometry results were observed in CL LVCP tissues (Additional file 1: Fig. S7a–e). Moreover, the cytotoxic CD8^+^ T cells and Iba1^+^ microglia were increased in the LVCP in stroke Veh-control brains at 24 h Rp, compared with Sham brain (Fig. 5f and g, *p* < 0.01). Pharmacological inhibition of SPAK–NKCC1 cascade suppressed CD8^+^ T and Iba1^+^ infiltration (Fig. 5f and g, *p* < 0.05), consistent with the preserved ChP blood–CSF barrier integrity shown in Fig. 4. CL LVCP exhibited similar changes of CD8^+^ cells under above conditions (Additional file 1: Fig. S7f and g). Taken together, these data further show that dysregulation of SPAK–NKCC1 complex plays a role in the ChP blood–CSF barrier integrity and immune cell infiltration.

### Oxidative stress is a mechanism in stimulating SPAK–NKCC1 cascade and induces apoptosis of cultured CPECs

**Fig. 6:**
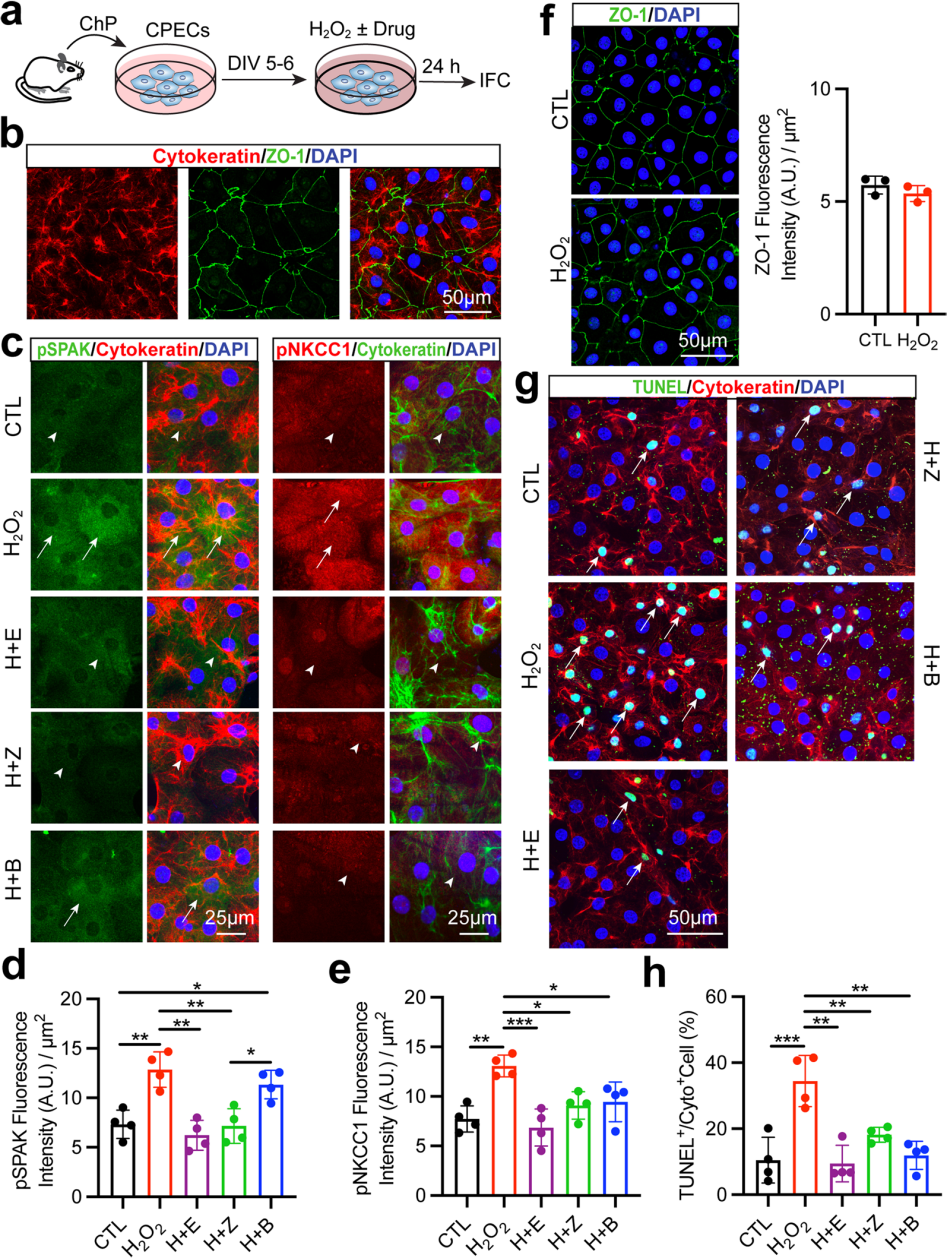
H_2_O_2_-mediated stimulation of SPAK–NKCC1 cascade and CPECs apoptosis are blocked by ZT-1a and BMT. **a** Experiment protocol. **b** Representative confocal images of cultured CPECs stained for cytokeratin and ZO-1. **c** Representative confocal immunostaining images of pSPAK/pNKCC1 and epithelial marker cytokeratin in CPECs. Cells were exposed to either normal DMEM/F12 medium (CTL), 200 µM H_2_O_2_, 200 µM H_2_O_2_ + 1 µM Ebselen (H + E), 200 µM H_2_O_2_ + 1 µM ZT-1a (H + Z) or 200 µM H_2_O_2_ + 10 µM BMT (H + B) in the culture medium for 24 h. Arrowheads: low level expression of pSPAK or pNKCC1. Arrows: elevated pSPAK or pNKCC1 expression. **d** and **e** Summary data of pSPAK or pNKCC1 fluorescence intensity. Data are mean ± SD (*n* = 4). *p < 0.05, **p < 0.01, ***p < 0.001. One-way ANOVA. **f** Representative confocal image of ZO-1 and summary, data are mean ± SD (*n* = 3). **g** Representative image of TUNEL^+^ CPECs. Arrows: TUNEL^+^/cytokeratin^+^ cells. **h** Quantitative analysis of TUNEL^+^ cell counts. Data are mean ± SD (*n* = 4). **p < 0.01, ***p < 0.001. One-way ANOVA

The underlying mechanisms in which ischemic stroke triggers activation of SPAK–NKCC1 complex in ChP remains undefined. It was reported that ROS stimulate SPAK phosphorylation and causes Claudin-18 disruption in alveolar epithelium cell following hyperoxia insult [44]. To determine whether the ROS-mediated mechanism upregulates SPAK–NKCC1 cascade phosphorylation activity in the ChP, we established an in vitro model of primary cultures of CPECs which expressed abundant epithelial cell cytoskeletal protein cytokeratin as well as TJ ZO-1 (Fig. 6b). To mimic oxidative stress, we exposed CPECs to H_2_O_2_ for 24 h and measured H_2_O_2_-induced activation of SPAK–NKCC1 signaling pathway with immunostaining (Fig. 6a). H_2_O_2_-treated CPECs significantly stimulated SPAK–NKCC1 phosphorylation activation, but the CPECs treated with H_2_O_2_ plus the ROS scavenger Ebselen for 24 h failed to activate SPAK–NKCC1 signaling (Fig. 6c–e, *p* < 0.01). Exposing CPECs to H_2_O_2_ combined with ZT-1a, but not BMT, downregulated pSPAK protein expression (Fig. 6c and d, *p* < 0.01). Moreover, H_2_O_2_ plus ZT-1a or BMT suppressed oxidative stress-induced pNKCC1 protein expression (Fig. 6c and e, *p* < 0.05). We next assessed whether oxidative stress induced TJ ZO-1 damage, as shown in Fig. 6f, there were no differences in ZO-1 staining intensity in the control and H_2_O_2_-treated CPECs, which is consistent with our in vivo data. Moreover, H_2_O_2_-treated CPECs significantly increased TUNEL^+^ apoptotic cell counts (Fig. 6g and h, *p* < 0.001). In contrast, exposing CPEC cultures to ZT-1a or BMT in combination with H_2_O_2_ failed to induce apoptotic death, which is similar to H_2_O_2_ scavenger Ebselen effects (Fig. 6g and h, *p* < 0.01). In summary, oxidative stress activated the SPAK–NKCC1 complex and induced cell apoptosis in cultured CPECs.

### Pharmacological inhibition of the SPAK–NKCC1 signaling pathway reduced H_2_O_2_-mediated ion influx and neuronal death

**Fig. 7:**
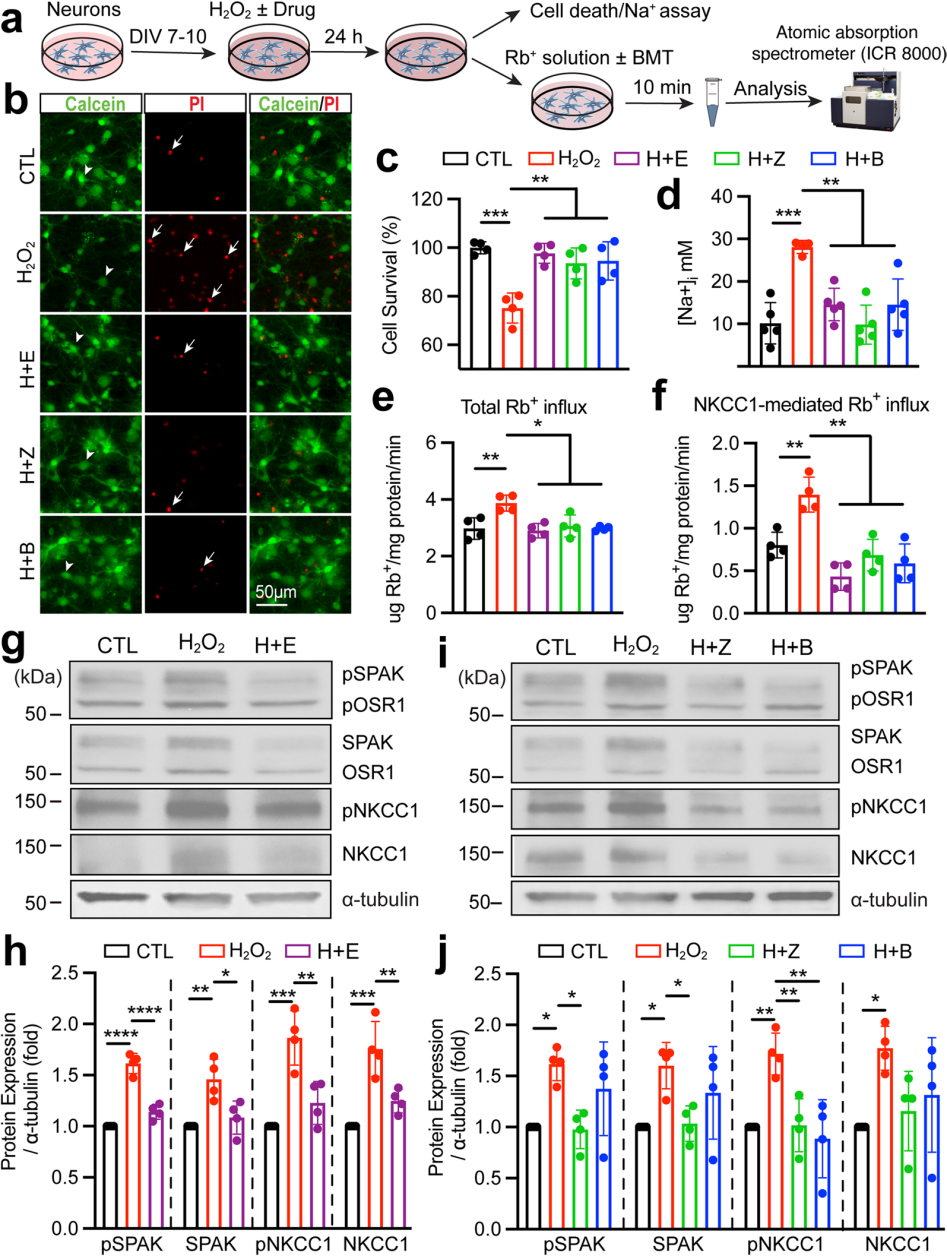
H_2_O_2_-mediated activation of SPAK–NKCC1 cascade-mediated Na^+^, K^+^ (Rb^+^) influx and cell death in cultured neurons. **a** Experiment protocol. **b** Representative fluorescent images of primary cortical neuron cultures. Cells were stained with Calcein-AM and propidium iodide (PI) at 24 h post-exposure either to control (CTL), 20 µM H_2_O_2_, 20 µM H_2_O_2_ + 100 nM Ebselen (H + E), 20 µM H_2_O_2_ + 1 μM ZT-1a (H + Z), or 20 µM H_2_O_2_ + 10 μM BMT (H + B). Arrowheads: live neurons were stained with Calcein-AM (green); arrows: dead neurons were stained with PI (Red). **c** Neuronal survival rates. Values are calculated as the number of live cells divided by the total number of live plus dead cells, multiplied by 100. Data are expressed as mean ± SD (*n* = 4), **p < 0.01, ***p < 0.001. **d** Representative intracellular Na^+^ [Na^+^]_i_ concentration using Na^+^-sensitive dye SBFI/AM at 24 h post-exposure of CTL and H_2_O_2_ insult ± drug treatment conditions. Values are mean ± SD (*n* = 4–5), **p < 0.01, ***p < 0.001. **e** and **f** Total Rb^+^ (K^+^) influx and NKCC1-mediated Rb^+^ (K^+^) influx. Neuron cells were exposed to each condition for 24 h. Rb^+^ influx into cells under isotonic (310 mOsm, pH 7.4) solutions were assayed for 10 min and identified using ICR 8000. Values are mean ± SD (*n* = 4), *p < 0.05, **p < 0.01. **g** Representative immunoblots of primary cortical neuron cultures under conditions as in **b**. Cell lysates were subjected to SDS-PAGE and incubated with appropriate antibody. **h** Summary. Values are expressed as mean ± SD (*n* = 4), *p < 0.05, **p < 0.01, ***p < 0.001, ****p < 0.0001. **i** Representative immunoblots of primary cortical neuron cultures under conditions as in **b**. **j** Summary. Data are mean ± SD (*n* = 4), *p < 0.05, **p < 0.01. One-way ANOVA

To further strengthen our conclusion, we also assessed whether the same mechanism of H_2_O_2_-mediated activation of the SPAK–NKCC1 signaling pathway occurs in primary cortical neurons (Fig. 7a). In the control conditions, the majority of living cortical neurons were stained with Calcein-AM (green) and less PI (red, Fig. 7b). H_2_O_2_ reduced the survival rate to 75%, significantly lower than the control group (Fig. 7b and c, *p* < 0.01). However, in the presence of H_2_O_2_ and Ebselen, the oxidative stress-induced neuronal death was prevented, and the survival rate was maintained at 98% (Fig. 7b and c, *p* < 0.01). H_2_O_2_ plus ZT-1a or BMT failed to trigger H_2_O_2_-mediated neuronal death (Fig. 7b and c, *p* < 0.01). These results indicate that pharmacological inhibition of the SPAK–NKCC1 signaling pathway with ZT-1a or BMT is also protective against H_2_O_2_-induced neuronal death. We next examined the intracellular Na^+^ ([Na^+^]_i_) concentration and the impact of elevated NKCC1-mediated Rb^+^ (K^+^) influx in neurons in response to H_2_O_2_ insult. As predicted, exposure of neurons to H_2_O_2_ for 24 h significantly increased [Na^+^]_i_ by ~ 2.9 fold (from 10.2 ± 4.4 mM to 28.1 ± 1.3 mM, Fig. 7d, *p* < 0.001). In contrast, inhibition of SPAK with ZT-1a and NKCC1 with BMT prevented H_2_O_2_-mediated rise in [Na^+^]_i_ (9.9 ± 4.1 mM and 14.5 ± 5.4 mM, respectively, Fig. 7d, *p* < 0.01). Similarly, H_2_O_2_ scavenger Ebselen treatment also significantly prevented the H_2_O_2_-mediated rise in [Na^+^]_i_ by ~ 48% (Fig. 7d, *p* < 0.01). In addition, exposure of neurons to H_2_O_2_ for 24 h significantly increased the total Rb^+^ and NKCC1-mediated Rb^+^ influx (determined in the presence of 10 μM BMT during Rb^+^ assay) by ~ 23% and by ~ 41%, respectively (Fig. 7e and f, *p* < 0.01) in isotonic conditions (310 mOsm). H_2_O_2_ treatment in the presence of Ebselen for 24 h failed to stimulate total or NKCC1-mediated Rb^+^ influx (Fig. 7e and f, *p* < 0.05). We further assessed whether blockade of SPAK–NKCC1 signaling pathway activity with ZT-1a or BMT would prevent H_2_O_2_-mediated stimulation of the Rb^+^ influx in neuronal cells. As Fig. 7e and f shows that H_2_O_2_ in combination with ZT-1a or BMT effectively decreased the total Rb^+^ (by ~ 20% and ~ 23%, respectively, *p* < 0.05) as well as NKCC1-mediated Rb^+^ influx (by ~ 51% and ~ 58%, respectively, *p* < 0.01). Finally, we assessed protein expression changes with immunoblotting to prove that H_2_O_2_ stress indeed triggered SPAK–NKCC1 activation. As shown in Fig. 7g and h, H_2_O_2_ exposure triggered a significant increase in the expression of pSPAK/SPAK and pNKCC1/NKCC1 protein in neurons, compared to the control group (*p* < 0.01). The H_2_O_2_-mediated activation of SPAK–NKCC1 cascade pathway is indeed mediated by oxidative stress, since concurrently exposing neurons to H_2_O_2_ and Ebselen failed to trigger SPAK–NKCC1 signaling pathway activation and displayed no changes compare to control (Fig. 7g and h). Compared to the H_2_O_2_ insult group, exposure of neurons to H_2_O_2_ combined with ZT-1a (1 μM) significantly decreased H_2_O_2_-mediated stimulation expression of pSPAK/pNKCC1 protein as well as upregulation of SPAK protein (Fig. 7i and j, *p* < 0.05). In contrast, exposure of neurons to H_2_O_2_ in combination with BMT (10 μM) only decreased the expression of pNKCC1 protein (Fig. 7i and j, *p* < 0.01) and has no effect on upstream SPAK proteins compared to the H_2_O_2_-treated group. Taken together, these data show that H_2_O_2_ not only induced phosphorylation activation of the SPAK–NKCC1 signaling complex, NKCC1-mediated K^+^ and Na^+^ influx, but also upregulated SPAK and NKCC1 protein expression in cultured neurons, which collectively contributed to neuronal death.

## Discussion

### Significance of SPAK–NKCC1 complex proteins in ChP structure and function

The ChP being formed by cuboidal epithelial cells and surrounding a core of fenestrated capillaries are involved in the production of CSF [45], which plays a prominent role in the regulation of brain development, neuronal function, nutrient transport and removal of metabolites [10, 12]. The specialized CPECs, interconnected by TJ at the basement membrane, form the blood–CSF barrier between the capillaries and the CSF [46]. The NKCC1, expressed in the apical membrane of CPECs, transports salt and water, contributing approximately half of the CSF production [12]. SPAK, a master regulator of epithelial ion transporters and channels, is highly expressed and localized to the apical membrane of the CPECs [7, 35]. Phosphorylation of SPAK–NKCC1 cascade at the CPECs apical membrane results in hyperactive CSF secretion following intraventricular hemorrhage [7]. Stimulation of NKCC1 at the luminal membrane of BBB endothelial cells has also been reported to aggravate the initial edema formation and BBB disruption after ischemic stroke or TBI [47, 48]. Pharmacological inhibition or a genetic deficiency of NKCC1 decreases BBB breakdown, brain edema and retains TJ protein integrity [47], but the role of NKCC1 in the ChP blood–CSF barrier after ischemic stroke has received little attention. In this study, we showed that both the SPAK and NKCC1 proteins were phosphorylated, activated at the apical membrane of the CPECs at 24 h Rp after stroke. In addition, activation of the SPAK–NKCC1 cascade was correlated with the ChP blood–CSF barrier damage and dysfunction. Our new findings in this study demonstrate that stroke-induced activation of the SPAK–NKCC1 complex at the ChP is associated with barrier dysfunction, immune cell infiltration, activation of NF-κB and MMP9 signaling at the ChP.

### Pharmacological blockade of SPAK–NKCC1 complex is protective of ChP after ischemic stroke

SPAK can associate with the (R/K)FX(V/I) motif in the NKCC1 and phosphorylate its N terminus at Thr203, Thr207, and Thr212 amino acids, which play an important role in inflammation [7, 49]. SPAK integrates and transduces environmental stress signals, including NF-κB-dependent inflammatory signals [7]. The 5′-flanking region of the SPAK gene contains two transcriptional start sites, three transcription factor Sp1-binding sites, and one transcription factor NF-κB-binding site [49]. siRNA-mediated knockdown of NF-κB reduced SPAK protein expression in human intestinal cell line Caco2-BBE [49]. Increased expression of MMP9 through the NF-κB signaling pathway occurred in angiostrongyliasis meningoencephalitis, leading to Claudin-5 degradation and alteration of the blood–CSF barrier permeability [50]. We speculate that increased SPAK activation may exacerbate the inflammatory environment and damage of Claudin-1 and Claudin-5. In our study, we detected increased NF-κB pathway activation and MMP9 protein expression in ChP from ischemic stroke brains, which likely contributes to damage to the ChP barrier integrity. Both the SPAK inhibitor ZT-1a and the well-established NKCC1 inhibitor BMT blocked SPAK and NKCC1 activation, reduced MMP9 protein expression and exhibited preservation of TJ Claudin-1 and Claudin-5 in the ChP, suggesting that inhibition of SPAK with ZT-1a may suppress its upstream NF-κB activation. This is supported by findings that BMT treatment or NKCC1 deficiency suppressed upregulation of MMP9 expression and prevented the BBB TJ protein degradation in TBI [47]. We also detected that ischemic stroke caused loss of the ChP blood–CSF barrier restriction of plasma albumin in the fenestrated ChP vasculature and increased CPECs’ uptake of albumin. The underlying mechanisms require additional investigations because multiple mechanisms could be involved, including breakdown of the TJ at the ChP, increased transcytosis of albumin in CPECs, and/or elevation of albumin specific transport proteins, etc. [43, 51, 52].

### ChP SPAK–NKCC1 activation and immune cell infiltration in ischemic stroke brain

The ChP structure is much simpler than BBB and is a gateway for leucocyte infiltration into the CNS [1]. Reducing blood supply to the LVCP caused the blood–CSF barrier to be disrupted as indicated by the increased [^3^H]-inulin entry into CSF and hippocampus [46]. Immune cells from the peripheral blood while under the influence of chemokines undergo adhesion, rolling, and diapedesis across the fenestrated capillary endothelium and pia mater of the ChP [53]. The invasion of lymphocytes through the ChP is a critical aspect of neuroinflammation following ischemic stroke, particularly due to its role in affecting initial infarct volume and thus the eventual poor clinical outcome [11]. In this study, we detected that pathological stimulation of the SPAK–NKCC1 cascade after ischemic stroke causes the ChP blood–CSF barrier integrity dysfunction and elevated infiltration of T cells, CD11b^+^CD45^+^/Ly6G^+^ neutrophils and CD11b + CD45^hi^ myeloid cells into both the CL and IL ChP, contributing to neuroinflammation. Inhibition of SPAK–NKCC1 complex not only preserved the barrier integrity, but also attenuated immune cell infiltration, emerging SPAK–NKCC1 signaling cascade as a therapeutic target for attenuating ChP dysfunction and brain inflammation after stroke.

### H_2_O_2_ stimulates expression and activation of SPAK–NKCC1 signaling pathway in CPECs and neurons

Free radical and ROS play important roles in ischemic reperfusion injury. Oxidative damage led to disruption of the blood–CSF barrier in sepsis-associated encephalopathy [54]. The underlying cellular and molecular mechanisms are not completely understood. A recent study shows that ROS-mediated upregulation of pSPAK in alveolar epithelium cells is linked to loss of TJ Claudin-18 protein, knockdown of SPAK preserved the alveolar epithelium barrier integrity after hyperoxic stress [44]. Moreover, manganese, oxidants and NO donors have been reported to increase the oxidation and nitration of NKCC1 protein and upregulation of total and phosphorylated NKCC1 protein expression in cultured astrocytes [55]. Those research findings suggest that oxidative stress plays an important role in regulating SPAK–NKCC1 complex expression and post-translational modulation. However, it is unknown whether stimulation of SPAK–NKCC1 complex in the CPECs or neurons is regulated by oxidative stress. Now, we provided direct evidence that H_2_O_2-_mediated activation of the SPAK–NKCC1 signaling pathway stimulated NKCC1-mediated K^+^ and Na^+^ influx and cell damage. The oxidative stress-mediated changes can be blocked by Ebselen, which is an organoselenium compound with glutathione peroxidase (GPx)-like, thiol-dependent, hydroperoxide reducing activity [56]. In addition, Ebselen showed neuron protective effects in our study, which is consistent with previous reports about its antioxidant properties [57, 58]. Both ZT-1a and BMT alleviated damages induced by H_2_O_2_ in both CPECs and neurons. Together, ROS trigger SPAK–NKCC1 activation which led to intracellular ion dysregulation as well as apoptosis cell death in both CPECs and neurons.

## Conclusions

**Fig. 8:**
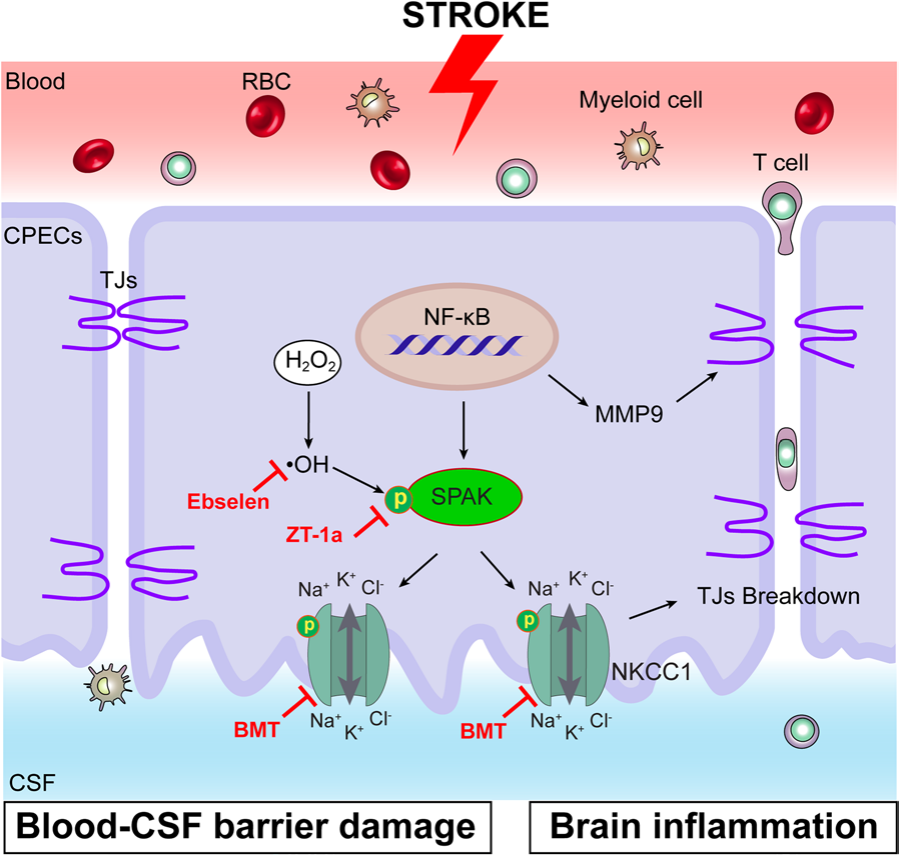
Schematic summary of stroke-induced dysregulation of the blood–CSF barrier via SPAK–NKCC1 signaling pathway. Ischemic stroke activation of SPAK–NKCC1 cascade in the ChP. Overstimulation of SPAK–NKCC1 signaling pathway activity leads to TJ damage, blood–CSF barrier dysfunction, and immune cell infiltration at the ChP. Ischemic reperfusion-mediated production of ROS can directedly activate SPAK–NKCC1 cascade and MMP9 protein expression with NF-κB activation as an upstream mechanism. Pharmacological inhibition of the SPAK–NKCC1 cascade attenuates blood–CSF barrier damage and brain inflammation

The integrity of ChP blood–CSF barrier plays a crucial role in maintaining CNS homeostasis. Stroke-induced damage of CPECs and TJ structures alters the ChP blood–CSF barrier function and the CNS neuroinflammation responses. As shown in Fig. 8, we report here that the stroke triggered stimulation of the SPAK–NKCC1 complex, NF-κB cascade as well as MMP9 protein expression in the CPECs, which is associated with degradation of TJ proteins and dysfunction of the ChP blood–CSF barrier. H_2_O_2_-mediated oxidative stress directly stimulated the activation of the SPAK–NKCC1 complex through protein phosphorylation in cultured CPECs and caused apoptosis. These findings suggest that stroke-induced stimulation of the ChP SPAK–NKCC1 cascade is in part mediated by oxidative stress. Pharmacological blockade of the SPAK–NKCC1 cascade has therapeutic potential for protection of the ChP blood–CSF barrier integrity and reducing CPECs damage as well as immune cell infiltration.

## Supplementary Information

**Additional file 1.** Supplemental file.

**Additional file 2.** Raw Immunoblot Images.

## Data Availability

Supporting data and information about used material are available from the corresponding author on reasonable request.

## Abbreviations

ChP: Choroid plexus
CSF: Cerebrospinal fluid
NKCC1: Na^+^–K^+^–Cl^−^ cotransporter 1
SPAK: Ste20-related proline-alanine-rich kinase
TJs: Tight junction proteins
CPECs: Choroid plexus epithelium cells
NF-κB: Nuclear factor kappa B
MMP9: Matrix metallopeptidase 9
CNS: Central nervous system
AD: Alzheimer’s disease
WNK: Kinase with no lysine (K)
OSR1: Oxidative stress-responsive kinase-1
TLR4: Toll-like receptor 4
BBB: Blood–brain barrier
H_2_O_2_: Hydrogen peroxide
BMT: Bumetanide
PI: Propidium iodide
TUNEL: Terminal deoxynucleotidyl-transferase-mediated dUTP nick-end labeling
tMCAO: Transient middle cerebral artery occlusion
CCA: Common carotid artery
ECA: External carotid artery
ICA: Internal carotid artery
MCA: Middle cerebral artery
Rp: Reperfusion
i.p.: Intraperitoneal injection
MAP2: Microtubule-associated protein 2
CL: Contralateral
IL: Ipsilateral
PFA: Paraformaldehyde
NGS: Normal goat serum
RT: Room temperature
DIV: Days in vitro
LVCP: Lateral ventricle choroid plexus
TBI: Traumatic brain injury
ROS: Reactive oxygen species
ROI: Region of interest
GLUT 1: Glucose transporter 1
