# Supplementary material for: Role of SPAK–NKCC1 signaling cascade in the choroid plexus blood–CSF barrier damage after stroke
Source: J Neuroinflammation. 2022 Apr 12;19:91. doi: 10.1186/s12974-022-02456-4 (PMC9006540; doi:10.1186/s12974-022-02456-4)

Role of SPAK-NKCC1 signaling cascade in the choroid plexus blood-CSF barrier damage after stroke

Jun Wang[1, 2], Ruijia Liu[2], Md Nabiul Hasan[2, 8], Sydney Fischer[2], Yang Chen[1, 2], Matt Como[3], Victoria M Fiesler[2], Mohammad Iqbal H. Bhuiyan[2, 8], Shuying Dong[2], Eric Li[2], Kristopher T Kahle[4], Jinwei Zhang[5], Xianming Deng[6], Arohan R Subramanya[7, 8], Gulnaz Begum[2, 8], Yan Yin[1]*, Dandan Sun[2, 8]*

[1]Department of Neurology, The Second Hospital of Dalian Medical University, Dalian, Liaoning, China
[2]Department of Neurology, University of Pittsburgh, Pittsburgh, PA, USA
[3]Pennsylvania State University, State College, PA, USA.
[4]Department of Neurosurgery, The Massachusetts General Hospital and Harvard Medical School, Boston, MA, USA
[5]Institute of Biomedical and Clinical Sciences, Medical School, College of Medicine and Health, University of Exeter, Hatherly Laboratory, Exeter, EX4 4PS, UK
[6]State Key Laboratory of Cellular Stress Biology, Innovation Center for Cell Signaling Network, School of Life Sciences, Xiamen University, Xiamen, Fujian, China
[7]Department of Medicine, Renal-Electrolyte Division, University of Pittsburgh School of Medicine, Pittsburgh, PA, USA
[8] Research Service, Veterans Affairs Pittsburgh Health Care System, Pittsburgh, PA, USA

Running title: **SPAK-NKCC1 complex in the blood-CSF barrier**

***Address correspondence to:**
Dandan Sun, M.D., Ph.D.
Department of Neurology
University of Pittsburgh
7016 Biomedical Science Tower 3, 3501 Fifth Ave.
Pittsburgh, PA 15260
Tel: (412) 624-0418, Fax: (412) 648-3321
E-mail address: sund@upmc.edu

Yan Yin, M.D., Ph.D.
Department of Neurology
The Second Hospital of Dalian Medical University
Dalian, Liaoning, China 116027
E-mail address: yanyin1208@126.com

SPAK-NKCC1 blot of isolated ChP in **Figure 2f**.

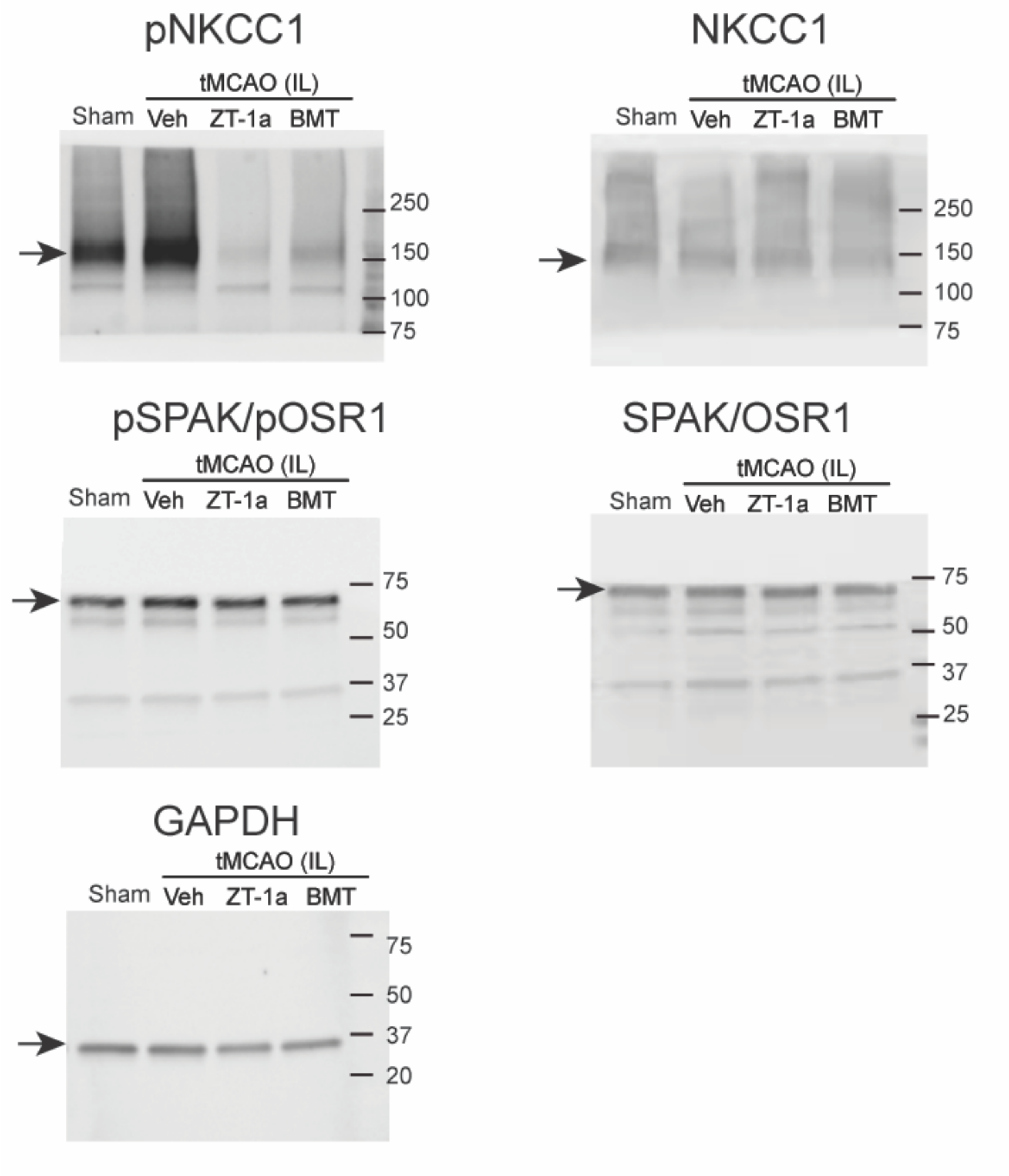

Clauin-1, Claudin-5 and ZO-1 blot of isolated ChP in **Figure 3e**.

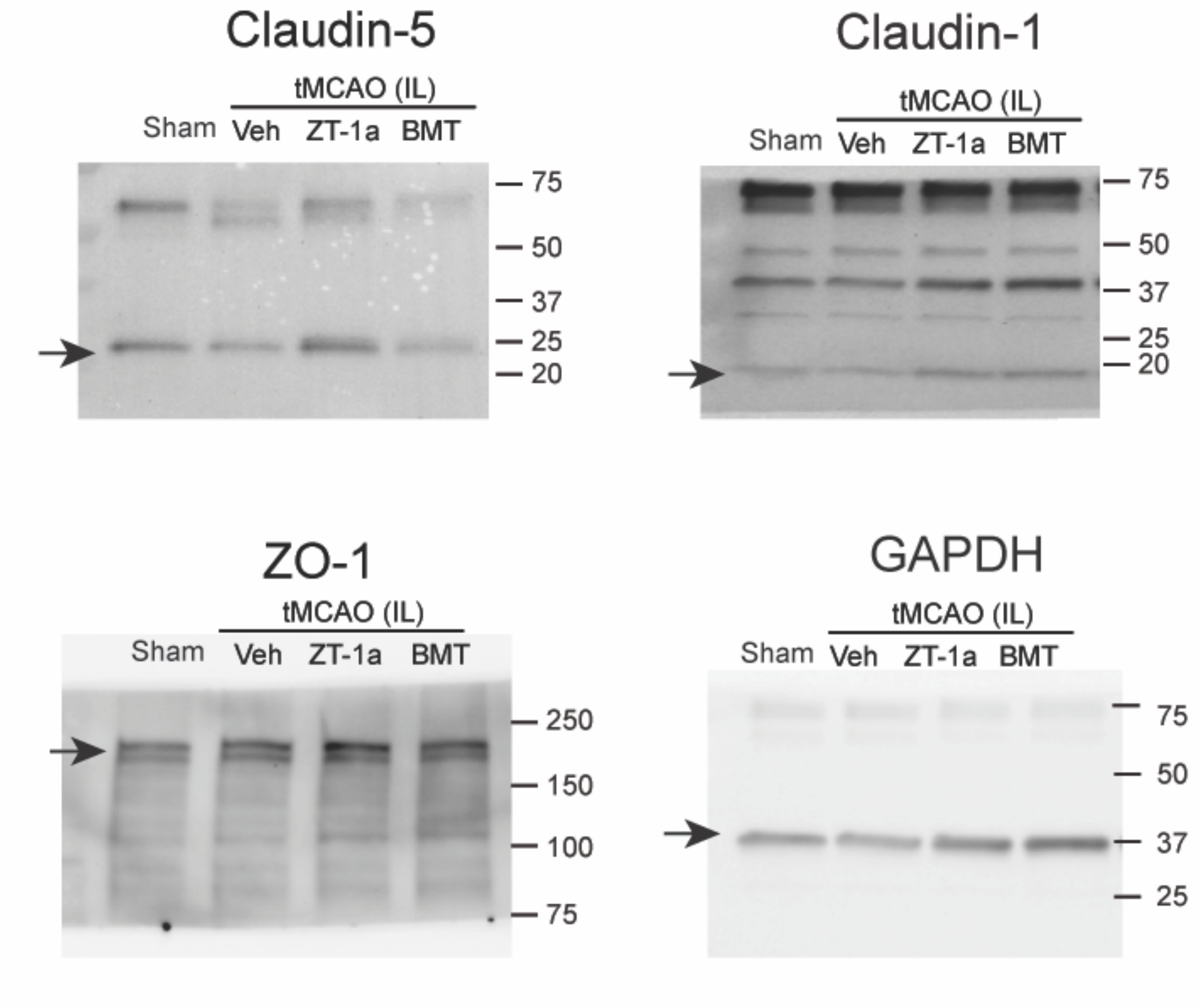

NF-κB and MMP9 blot of isolated ChP in **Figure 4e**.

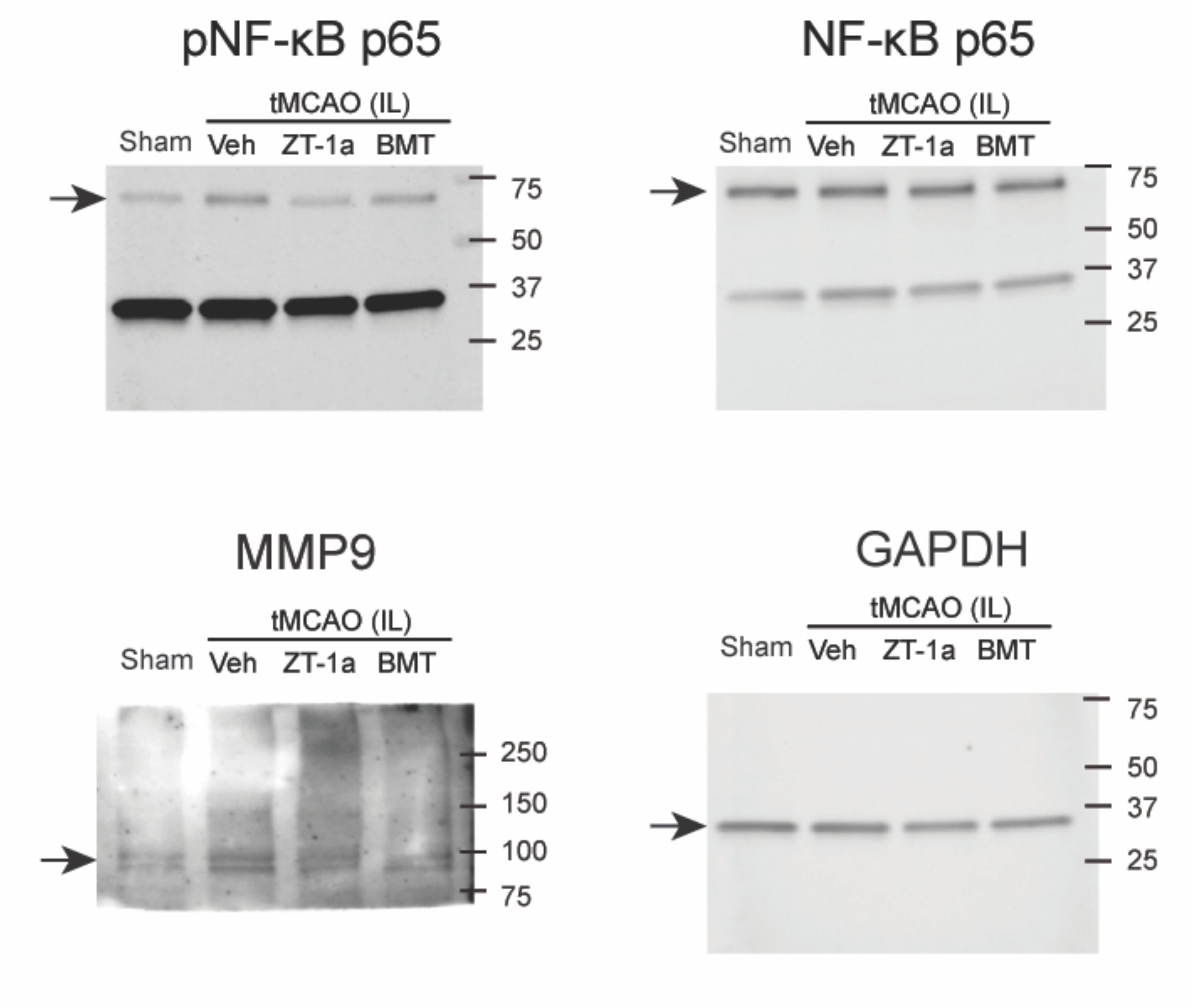

SPAK-NKCC1 blot of cultured neurons in **Figure 7g**.

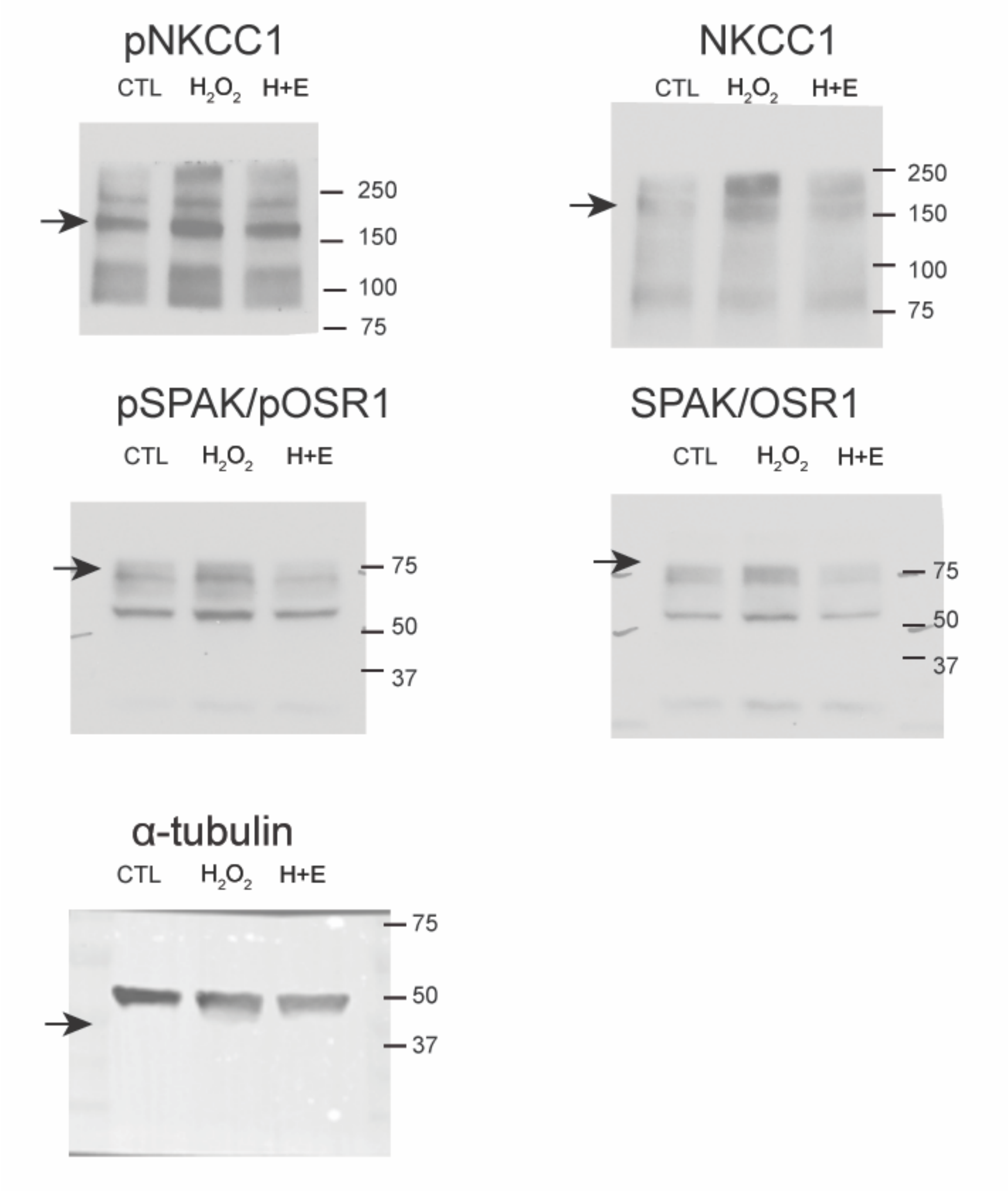

SPAK-NKCC1 blot of cultured neurons in **Figure 7i**.

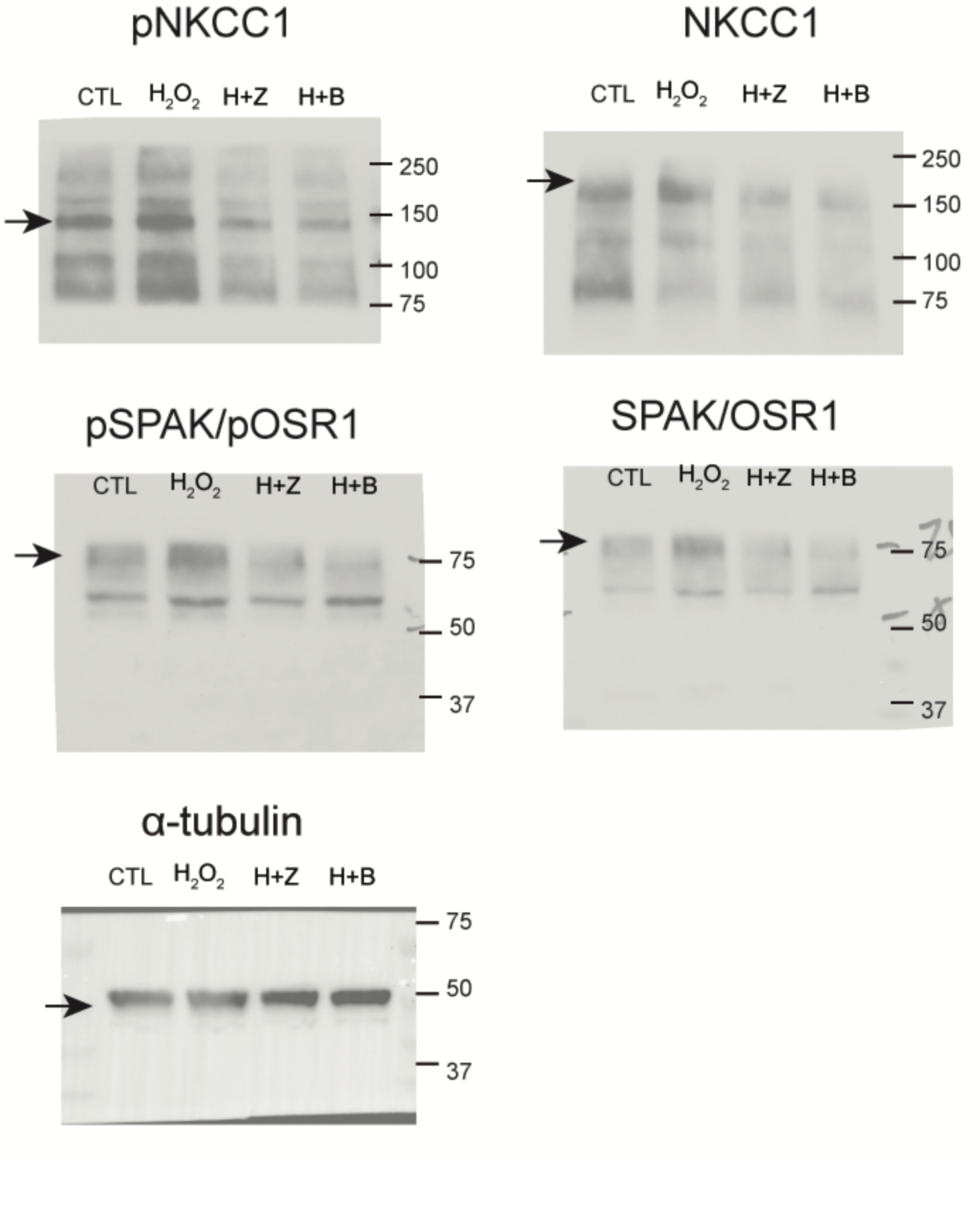

SPAK-NKCC1 blot of isolated ChP in **Supplementary Figure 2d**.

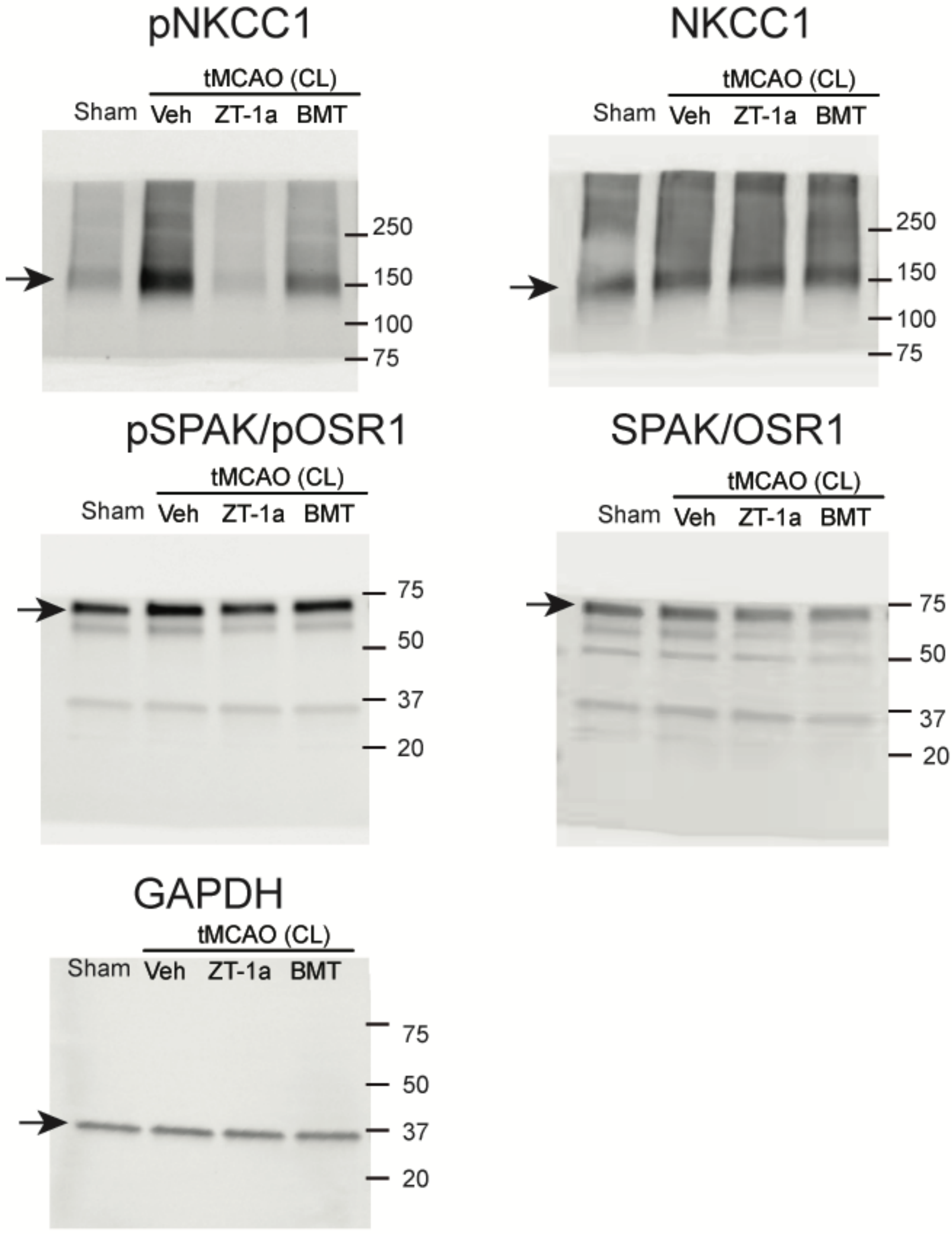

NF-κB and MMP9 blot of isolated ChP in **Supplementary Figure 5e**.

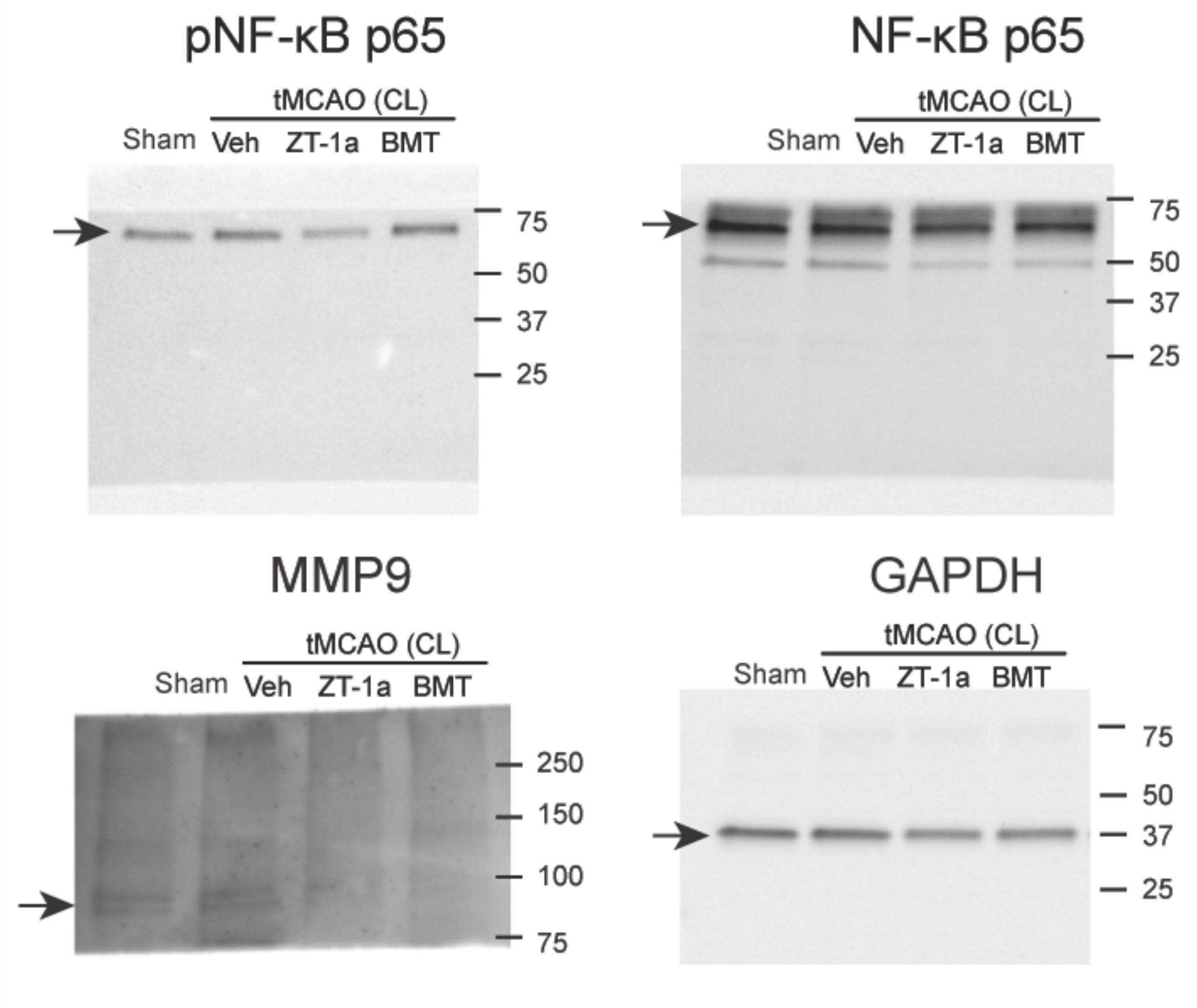

Supplement: Supplementary file 1 — Additional file 1. Supplemental file. [file 12974_2022_2456_MOESM1_ESM.pdf]
