# Supplementary material for: Role of SPAK–NKCC1 signaling cascade in the choroid plexus blood–CSF barrier damage after stroke
Source: J Neuroinflammation. 2022 Apr 12;19:91. doi: 10.1186/s12974-022-02456-4 (PMC9006540; doi:10.1186/s12974-022-02456-4)
Supplement: Supplementary file 2 — Additional file 2. Raw Immunoblot Images. [file 12974_2022_2456_MOESM2_ESM.pdf]

Role of SPAK-NKCC1 signaling cascade in the choroid plexus blood-CSF barrier damage after stroke

Jun Wang[1, 2], Ruijia Liu[2], Md Nabiul Hasan[2,8], Sydney Fischer[2], Yang Chen[1, 2], Matt Como[3], Victoria M Fiesler[2], Mohammad Iqbal H. Bhuiyan[2,8], Shuying Dong[2], Eric Li[2], Kristopher T Kahle[4], Jinwei Zhang[5], Xianming Deng[6], Arohan R Subramanya[7, 8], Gulnaz Begum[2,8], Yan Yin[1]*, Dandan Sun[2, 8]*

[1]Department of Neurology, The Second Hospital of Dalian Medical University, Dalian, Liaoning, China
[2]Department of Neurology, University of Pittsburgh, Pittsburgh, PA, USA
[3]Pennsylvania State University, State College, PA, USA.
[4]Department of Neurosurgery, The Massachusetts General Hospital and Harvard Medical School, Boston, MA, USA
[5]Institute of Biomedical and Clinical Sciences, Medical School, College of Medicine and Health, University of Exeter, Hatherly Laboratory, Exeter, EX4 4PS, UK
[6]State Key Laboratory of Cellular Stress Biology, Innovation Center for Cell Signaling Network, School of Life Sciences, Xiamen University, Xiamen, Fujian, China
[7]Department of Medicine, Renal-Electrolyte Division, University of Pittsburgh School of Medicine, Pittsburgh, PA, USA
[8] Research Service, Veterans Affairs Pittsburgh Health Care System, Pittsburgh, PA, USA

Running title: **SPAK-NKCC1 complex in the blood-CSF barrier**

***Address correspondence to:**
Dandan Sun, M.D., Ph.D.
Department of Neurology
University of Pittsburgh
7016 Biomedical Science Tower 3, 3501 Fifth Ave.
Pittsburgh, PA 15260
Tel: (412) 624-0418, Fax: (412) 648-3321
E-mail address: sund@upmc.edu

Yan Yin, M.D., Ph.D.
Department of Neurology
The Second Hospital of Dalian Medical University
Dalian, Liaoning, China 116027
E-mail address: yanyin1208@126.com

Supplementary Table 1. List of antibodies used for immunofluorescence staining (IF), Western blotting (WB) and flow cytometry.

Antibody	Host	Dilution	Company	Catalog No.	Application
SPAK	Rabbit	1:200	Cell Signaling Technology	2281	
pSPAK	Rabbit	1:200	EMD Millipore	07-2273	
NKCC1	Rabbit	1:200	Abcam	ab59791	
pNKCC1	Rabbit	1:200	EMD Millipore	ABS1004	
pT58 NCC (pThr211 NKCC1)	Rabbit	1:500	N/A	N/A	
ZO-1	Rabbit	1:200	Invitrogen	40-2200	IF
Iba1	Rabbit	1:200	Wako Bioproducts	019-19741	
Claudin-1	Rabbit	1:200	Invitrogen	51-9000	
Claudin-5	Mouse	1:200	Invitrogen	35-2500	
Cytokeratin	Mouse	1:200	Sigma	C2562	
MAP2	Mouse	1:200	EMD Millipore	MAB3418	
CD8	Rat	1:100	Invitrogen	14-0081-82	
Albumin	Rabbit	1:200	Abcam	ab19196	
GLUT1	Mouse	1:100	Abcam	ab40084	
SPAK/OSR1	Rabbit	1:300	N/A	N/A	
pSPAK/pOSR1	Rabbit	1:300	N/A	N/A	
NKCC1 (T4)	Mouse	1:3000	DSHB	T4	
pNKCC1	Rabbit	1:300	N/A	N/A	
pNF-κB p65	Rabbit	1:500	Cell Signaling Technology	3031S	
NF-κB p65	Rabbit	1:1000	Santa Cruz	SC-372	WB
MMP9	Rabbit	1:500	Abcam	ab283575	
GAPDH	Rabbit	1:5000	Cell Signaling Technology	2118S	
ZO-1	Rabbit	1:500	Invitrogen	40-2200	
Claudin-1	Rabbit	1:500	Invitrogen	51-9000	
Claudin-5	Rabbit	1:500	Invitrogen	35-2500	
α-tubulin	Mouse	1:5000	Invitrogen	138000	
BUV395-CD11b	Rat	250	BD Biosciences	56353	
APC-CD45	Rat	250	BioLegend	103111	
PerCP/Cy5.5-Ly6G	Rat	250	BioLegend	127615	
PE-Cy7-Ly-6C	Rat	250	BD Biosciences	560593	Flow Cytometry
BV421-CD3	Armenian Hamster	250	BioLegend	100336	
FITC-CD206	Rat	250	BioLegend	141704	
PE-Ym-1	Rabbit	2500	Abcam	Ab211621	

Supplementary Figure 1

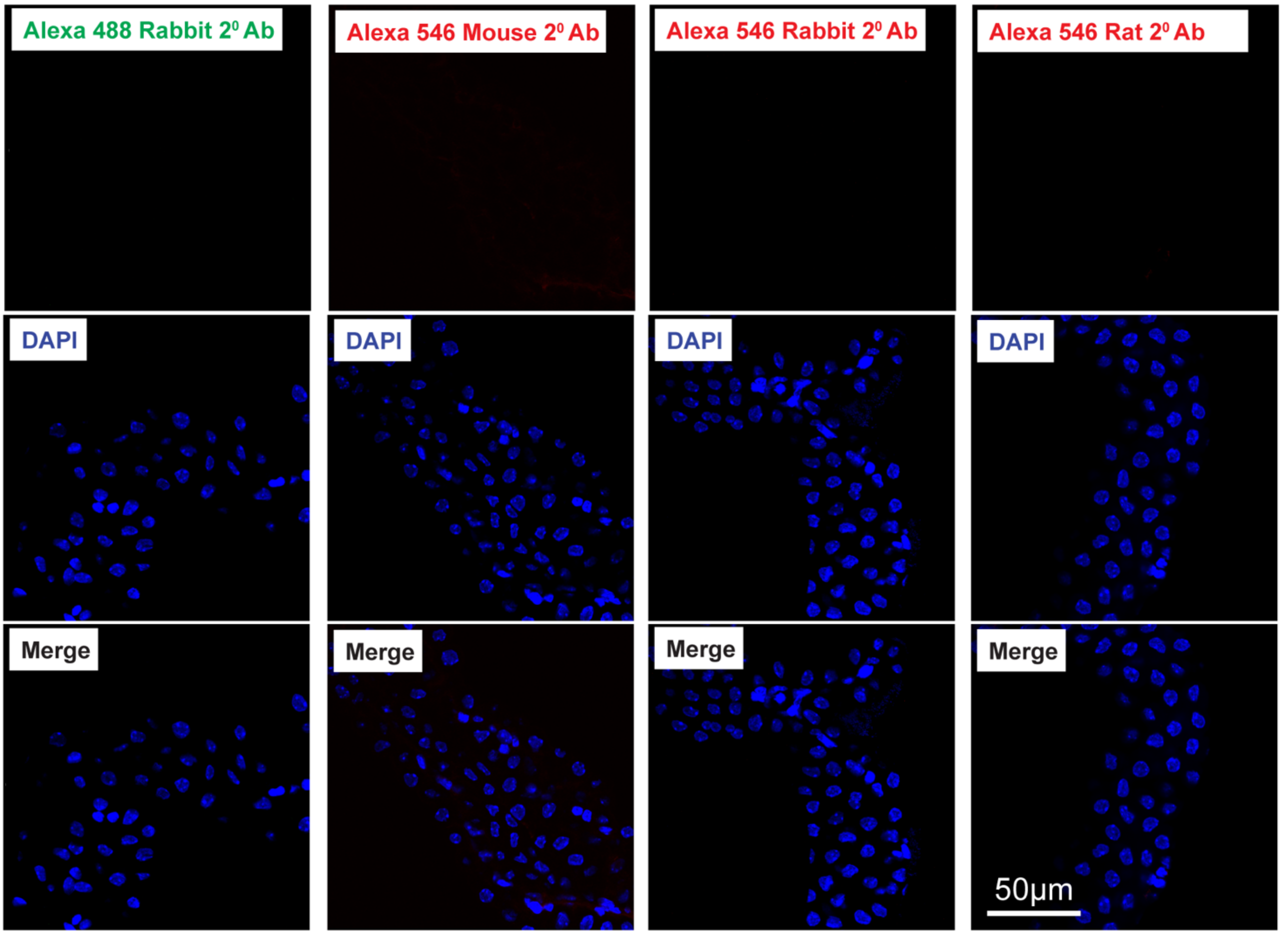

Supplementary Figure 1. Immunostaining negative controls.

Representative images of secondary antibody staining in the mouse LVCP with the following anibodies: Goat anti-rabbit Alexa 488, Goat anti-mouse Alexa 546, Goat anti-rabbit Alexa 546 and Goat anti-rat Alexa 546 at 1:200, images were taken under a 40x oil-immersion objective with identical settings.

Supplementary Figure 2

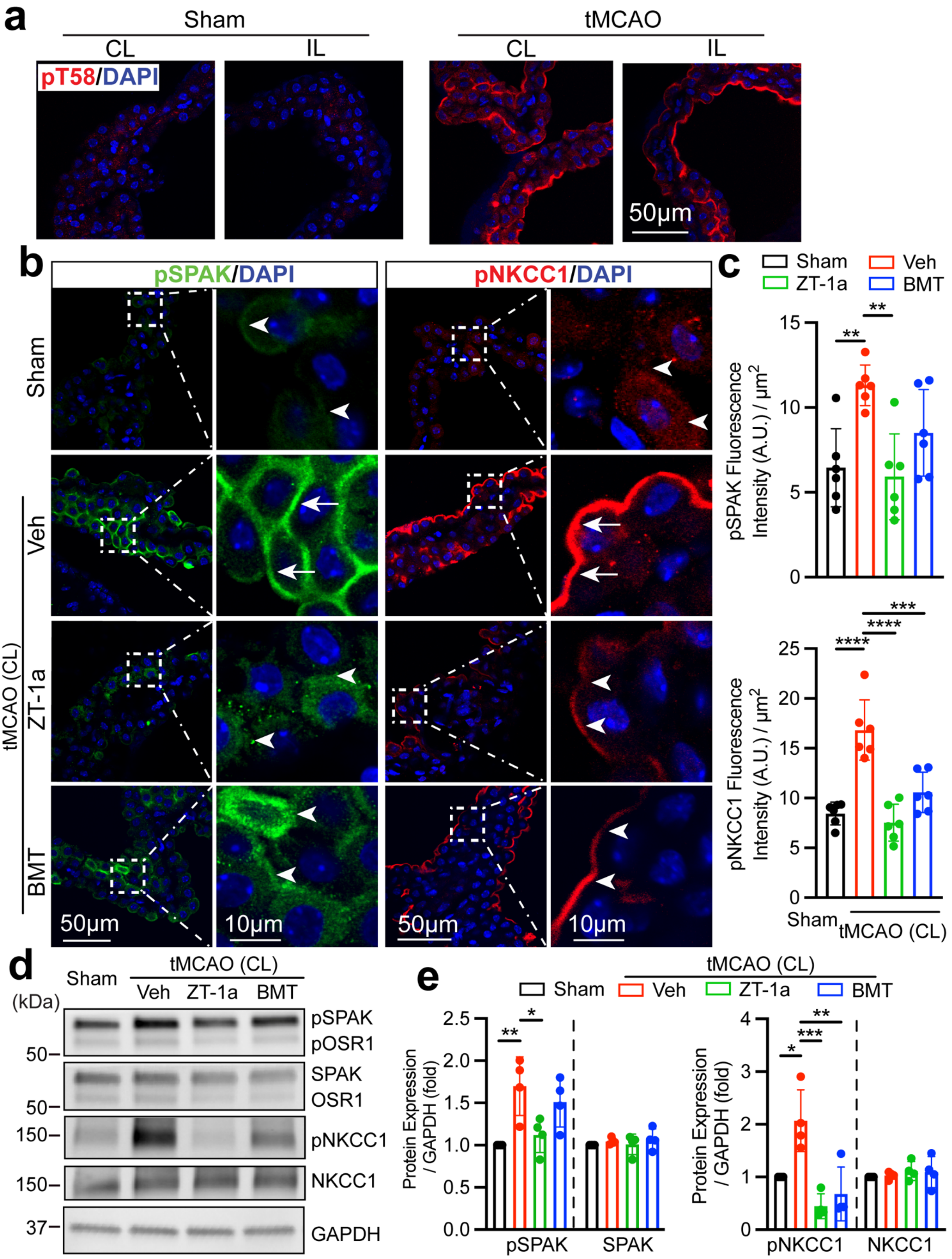

Supplementary Figure 2. Activation of SPAK-NKCC1 complex in the choroid plexus after ischemic stroke.

a. Representative immunostaining images of a phosphorylated species (active) of pT58 in Sham or stroke ChP. **b.** Representative immunofluorescent images of pSPAK and pNKCC1 staining of lateral ventricle choroid plexus (LVCP) in the contralateral (CL) hemispheres in Sham, stroke Veh-control, ZT-1a or BMT-treated brains. Arrowheads: low level of pSPAK or pNKCC1 expression. Arrows: elevated pSPAK or pNKCC1 expression. **c.** Quantification summary. Data are mean ± SD (n = 6, 4 male, 2 female), $^{**}p < 0.01$, $^{***}p < 0.001$, $^{****}p < 0.0001$. The Sham pSPAK and pNKCC1 data in panel **c** are the same as the Sham data presented in **Fig 2e**. **d.** Western blot analysis of SPAK-NKCC1 cascade expression in the CL ChP of LVCP in Sham, stroke Veh-control, ZT-1a- or BMT-treated mice at 24 h Rp after ischemic stroke. ChP tissue lysates were prepared and subjected to immunoblotting with the indicated antibodies. **e**. Immunoblot summary. Data are mean ± SD (n = 4, 2 male, 2 female). $^{*}p < 0.05$, $^{**}p < 0.01$, $^{***}p < 0.001$. One-way ANOVA.

Supplementary Figure 3

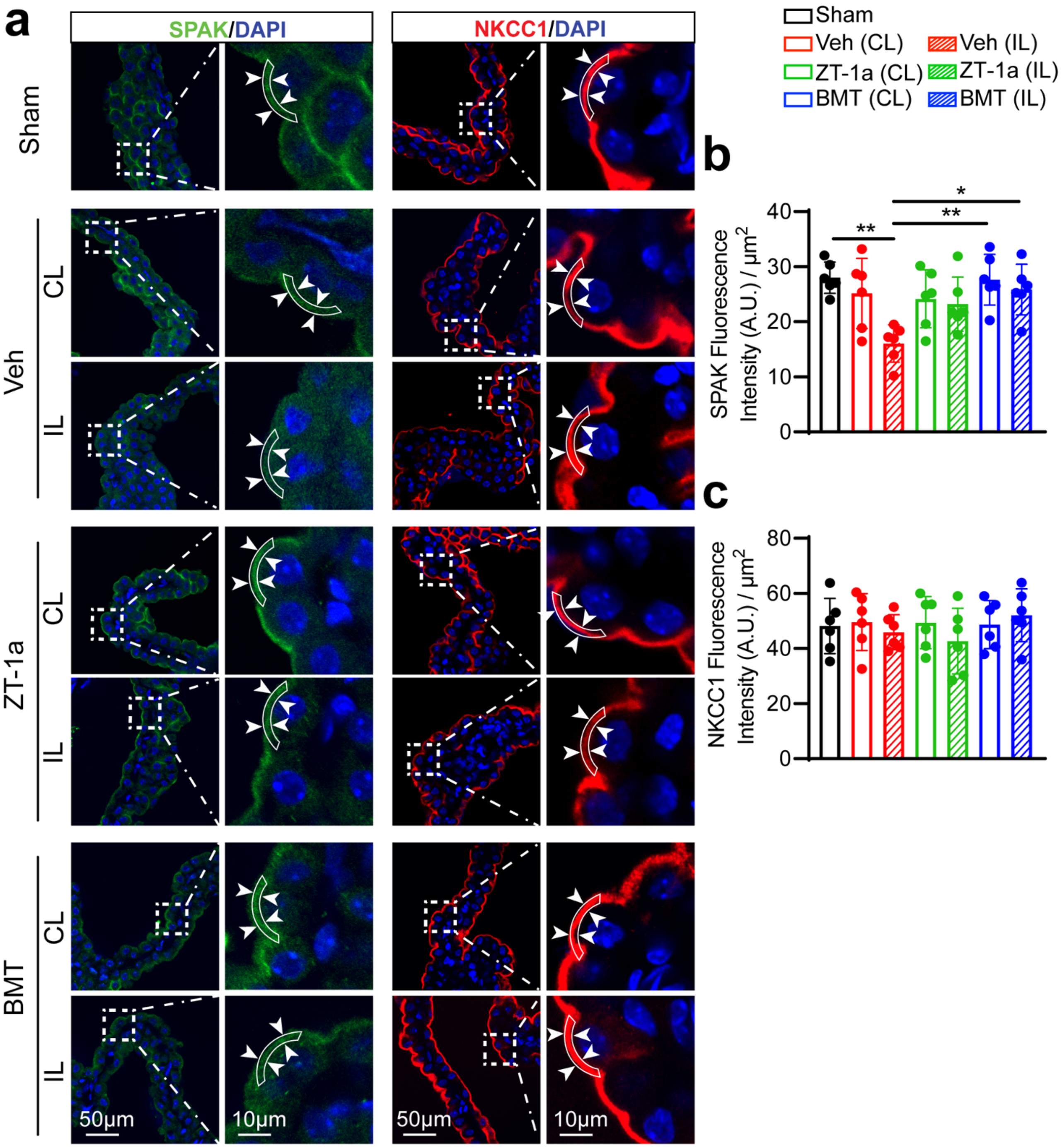

Supplementary Figure 3. Pharmacological blockade of SPAK-NKCC1 signaling cascade restored SPAK protein expression at the apical membrane of CPECs.

a. Representative confocal images of SPAK and NKCC1 protein expression in Sham or the Veh-, ZT-1a-, or BMT-treated post-stroke LVCP. Arrowheads: Expression of SPAK or NKCC1 at the apical

membrane of CPECs (Region of Interest). **b** and **c.** Summary data. Data are mean ±SD (n = 6, 4 male, 2 female). *$p < 0.05$, **$p < 0.01$. One-way ANOVA.

Supplementary Figure 4

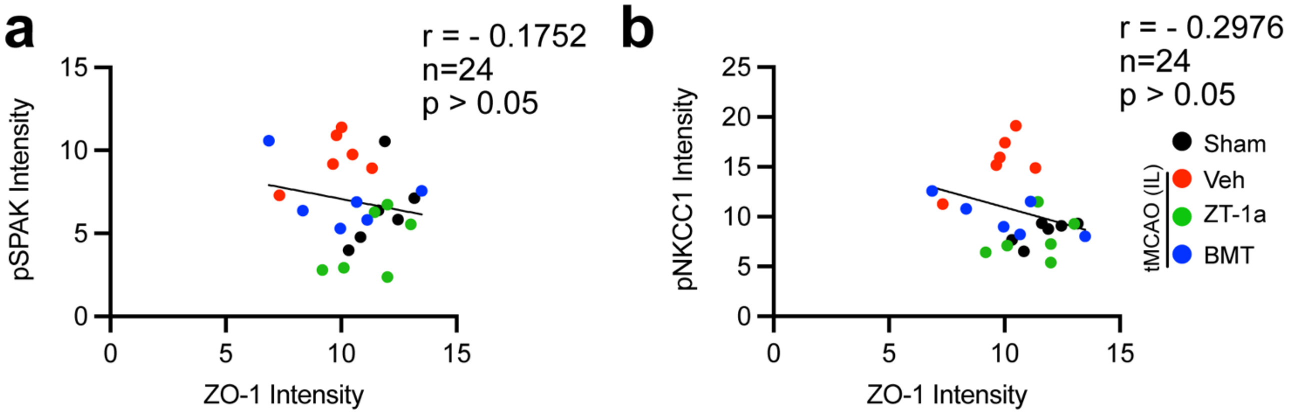

Supplementary Figure 4. Correlation between pSPAK, pNKCC1 and ZO-1 immunofluorescence staining intensity in the ChP.

a and **b.** Pearson correlation analysis between pSPAK or pNKCC1 and ZO-1 fluorescence intensity in ChP from the data presented in **Fig 3a** and **c**. n = 24.

Supplementary Figure 5

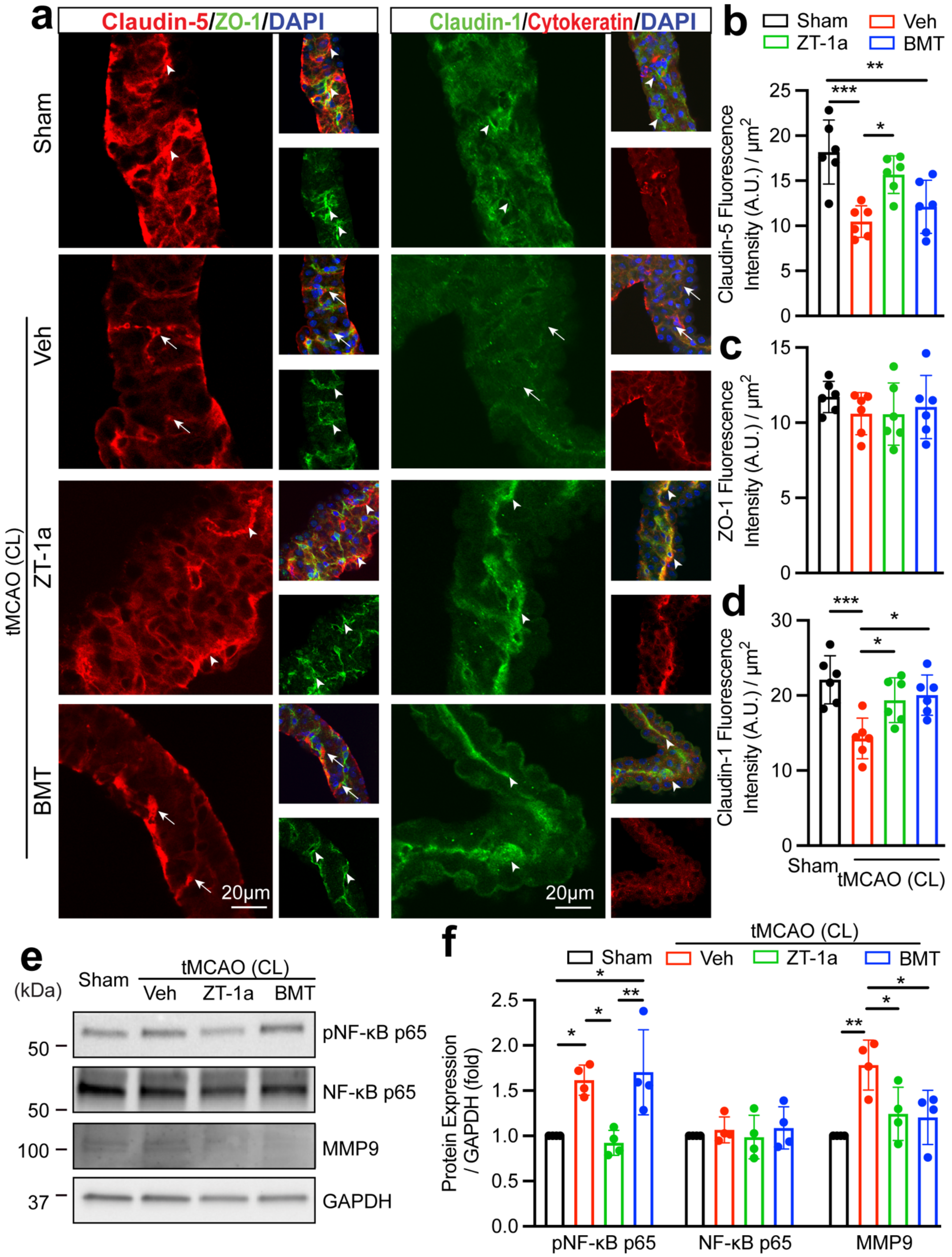

Supplementary Figure 5. Blocking SPAK-NKCC1 signal cascade preserved ChP tight junction integrity after ischemic stroke.

a. Representative confocal images of tight junction proteins (Claudin-5, ZO-1, Claudin-1) as well as the epithelial marker cytokeratin in the CL LVCP of Sham, Veh-control, ZT-1a- or BMT-treated stroke brains. Arrowheads: expression of Claudin-5, ZO-1 or Claudin-1. Arrows: reduced expression of Claudin-5 or Claudin-1. The Sham data in panel **b** - **d** are the same as the Sham data presented in **Fig 3b** - **d**. Data are represented as mean ± SD (n = 6, 4 male, 2 female). *$p < 0.05$, **$p < 0.01$, ***$p < 0.001$. **e.** Western blot analysis of pNF-κB p65, NF-κB p65 and MMP9 protein expression in the CL LVCP of Sham, Veh-control, ZT-1a- and BMT-treated stroke mice at 24 h Rp. ChP tissue lysates were prepared and subjected to immunoblotting with the indicated antibodies. **f.** Immunoblot quantitation. Data are means ± SD (n = 4, 2 male, 2 female). *$p < 0.05$, **$p < 0.01$. One-way ANOVA.

Supplementary Figure 6

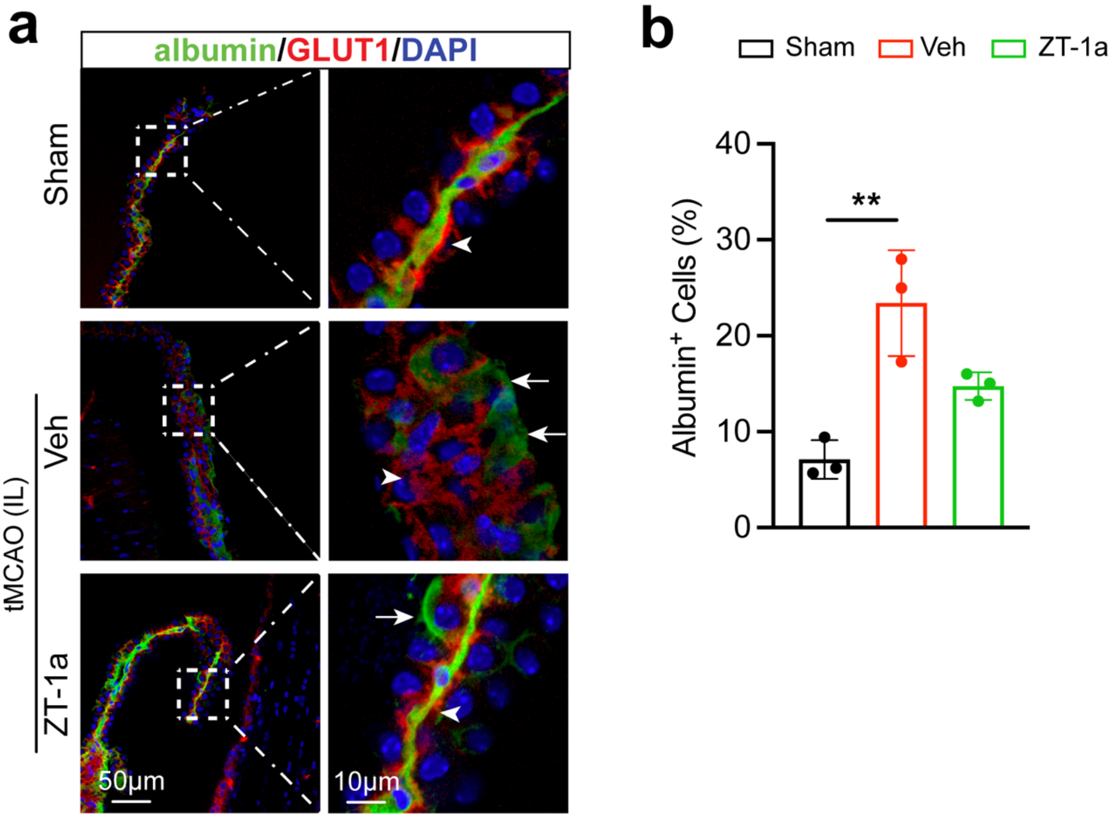

Supplementary Figure 6. Pharmacological inhibition of SPAK-NKCC1 signaling cascade attenuated the blood-CSF barrier permeability in stroke ChP.

a. Representative immunofluorescence confocal images of endogenous albumin protein as well as the endothelial marker glucose transporter 1 (GLUT 1) in the IL LVCP of Sham, Veh-control or ZT-1a-treated stroke brains at 24 h Rp. Arrowheads: fenestrated vessels. Arrows: albumin protein uptake by CPECs. **b.** Quantification summary. Data are mean ± SD (n = 3). **$p < 0.01$, One-way ANOVA.

Supplementary Figure 7

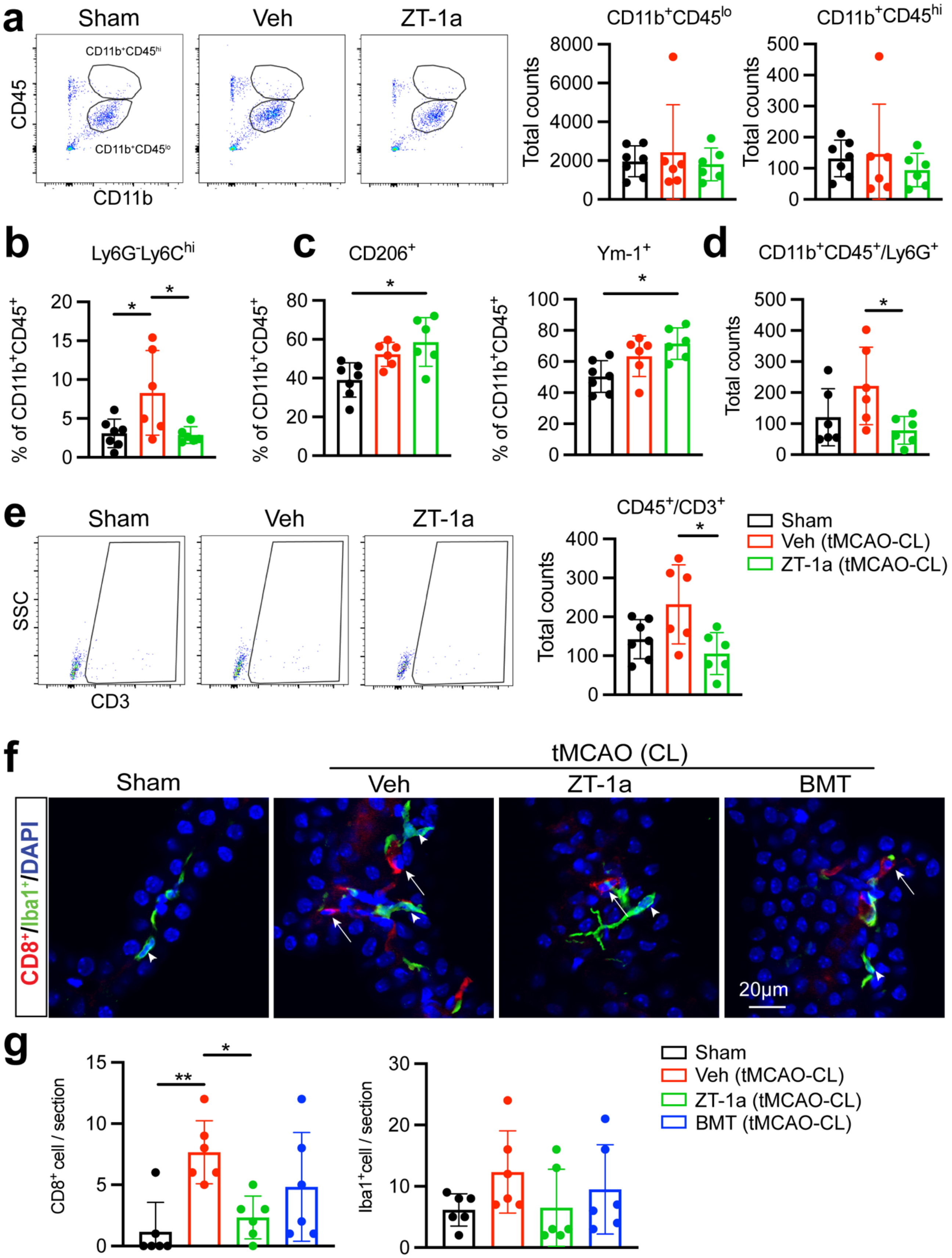

Supplementary Figure 7. Pharmacological inhibition of SPAK-NKCC1 signaling cascade reduced immune cell infiltration in the ChP.

a. Representative flow cytometric plots of $CD11b^{+}CD45^{lo}$ or $CD11b^{+}CD45^{hi}$ myeloid cells from the isolated CL ChP at 3 d post-surgery with quantification of total number of $CD11b^{+}CD45^{lo}$ or $CD11b^{+}CD45^{hi}$ myeloid cells in the ChP. **b** and **c.** Percentage of $CD11b^{+}CD45^{+}Ly6G^{-}Ly6C^{hi}$ or $CD206^{+}$ and Ym-1^{+} cells gated within $CD11b^{+}CD45^{+}$ cells. **d.** Total number of $CD11b^{+}CD45^{+}Ly6G^{+}$ neutrophils in the ChP. **e.** Representative flow cytometric plots and the total number of $CD3^{+}$ T cells in the ChP. Data are mean ± SD (n = 6-7). *$p < 0.05$. One-way ANOVA. **f.** Representative images of $CD8^{+}$ T cells (arrows) and $Iba1^{+}$ microglia cells (arrowheads) of CL LVCP in Sham, Veh-control, ZT-1a- or BMT-treated stroke mice at 24 h Rp. **g.** Summary. Data are mean ± SD (n = 6, 4 male, 2 female). *$p < 0.01$, **$p < 0.001$. One-way ANOVA.
